# Cerebrovascular Pathology in Hypertriglyceridemic APOB-100 Transgenic Mice

**DOI:** 10.3389/fncel.2018.00380

**Published:** 2018-10-25

**Authors:** Zsófia Hoyk, Melinda E. Tóth, Nikolett Lénárt, Dóra Nagy, Brigitta Dukay, Alexandra Csefová, Ágnes Zvara, György Seprényi, András Kincses, Fruzsina R. Walter, Szilvia Veszelka, Judit Vígh, Beáta Barabási, András Harazin, Ágnes Kittel, László G. Puskás, Botond Penke, László Vígh, Mária A. Deli, Miklós Sántha

**Affiliations:** ^1^Institute of Biophysics, Biological Research Centre, Hungarian Academy of Sciences, Szeged, Hungary; ^2^Institute of Biochemistry, Biological Research Centre, Hungarian Academy of Sciences, Szeged, Hungary; ^3^Laboratory of Functional Genomics, Core Facilities, Biological Research Centre, Hungarian Academy of Sciences, Szeged, Hungary; ^4^Department of Anatomy, Histology and Embryology, Faculty of Medicine, University of Szeged, Szeged, Hungary; ^5^Laboratory of Molecular Pharmacology, Department of Pharmacology, Institute of Experimental Medicine, Hungarian Academy of Sciences, Budapest, Hungary; ^6^Department of Medical Chemistry, Faculty of Medicine, University of Szeged, Szeged, Hungary

**Keywords:** apolipoprotein B-100, astroglia, blood-brain barrier, brain endothelial cell, cerebrovascular pathology, hypertriglyceridemia, P-glycoprotein, tight junction

## Abstract

Hypertriglyceridemia is not only a serious risk factor in the development of cardiovascular diseases, but it is linked to neurodegeneration, too. Previously, we generated transgenic mice overexpressing the human APOB-100 protein, a mouse model of human atherosclerosis. In this model we observed high plasma levels of triglycerides, oxidative stress, tau hyperphosphorylation, synaptic dysfunction, cognitive impairment, increased neural apoptosis and neurodegeneration. Neurovascular dysfunction is recognized as a key factor in the development of neurodegenerative diseases, but the cellular and molecular events linking cerebrovascular pathology and neurodegeneration are not fully understood. Our aim was to study cerebrovascular changes in APOB-100 transgenic mice. We described the kinetics of the development of chronic hypertriglyceridemia in the transgenic animals. Increased blood-brain barrier permeability was found in the hippocampus of APOB-100 transgenic mice which was accompanied by structural changes. Using transmission electron microscopy, we detected changes in the brain capillary endothelial tight junction structure and edematous swelling of astrocyte endfeet. In brain microvessels isolated from APOB-100 transgenic animals increased *Lox-1, Aqp4*, and decreased *Meox-2, Mfsd2a, Abcb1a, Lrp2, Glut-1, Nos2, Nos3, Vim*, and in transgenic brains reduced *Cdh2* and *Gfap-σ* gene expressions were measured using quantitative real-time PCR. We confirmed the decreased P-glycoprotein (ABCB1) and vimentin expression related to the neurovascular unit by immunostaining in transgenic brain sections using confocal microscopy. We conclude that in chronic hypertriglyceridemic APOB-100 transgenic mice both functional and morphological cerebrovascular pathology can be observed, and this animal model could be a useful tool to study the link between cerebrovascular pathology and neurodegeneration.

## Introduction

There is growing preclinical and clinical evidence that pathological changes at the level of the neurovascular unit (NVU), comprising all the cell types of cerebral microvessels and the surrounding neural tissue, lead to secondary neuronal injury and neurodegenerative diseases, including Alzheimer’s disease (AD) (Zhao et al., 2015). The key pathways of vascular dysfunction that are linked to neurodegenerative diseases include blood-brain barrier (BBB) breakdown, hypoperfusion-hypoxia and endothelial metabolic dysfunction (Zlokovic, 2008; Zhao et al., 2015). Morphologically the BBB is formed by the capillary endothelium, the basement membrane and the surrounding pericytes and astrocytic endfeet. The endothelial cells adhere tightly to one another, through junctional structures termed tight junctions (TJs), which restrict paracellular permeability of the BBB [3]. Several families of active influx and efflux transporters and transcytotic receptor systems regulate the exchange of small and large nutrients and metabolites across the BBB (Zlokovic, 2008). Both the structure and functions of the BBB are damaged in AD: the barrier function of TJs is impaired, the energy supply of neural cells is decreased, the entry of neurotoxic agents is elevated and the clearance of Aβ peptides is reduced (Zlokovic, 2008, 2011; Lyros et al., 2014; Di Marco et al., 2015; Zhao et al., 2015). Many studies suggest a direct link of atherosclerosis with not only vascular, but also AD dementia, although the relationship is still unclear (Nelson et al., 2016; Kapasi and Schneider, 2016). NVU and BBB pathologies have been increasingly investigated in genetic and other animal models of AD (Nicolakakis and Hamel, 2011), but there are very few models focusing on the link between atherosclerosis and dementia (Li et al., 2003; Lane-Donovan et al., 2016) or NVU changes.

Previously, we generated a mouse model of human atherosclerosis using transgenic mice overexpressing the human APOB-100 protein in different tissues such as the liver, heart and brain (Bjelik et al., 2006; Csont et al., 2007; Lénárt et al., 2012). Apolipoprotein B-100 (APOB-100) is a large, 512 kDa glycoprotein that circulates in the plasma as the major protein component of low density lipoprotein (LDL) and very low density lipoproteins (VLDL). The higher ratio of LDL and VLDL fractions compared to HDL in the blood of these transgenic animals is similar to the human plasma lipoprotein profile, therefore this mouse strain is more suitable to study the effects of hypercholesterinaemia and hypertriglyceridemia than the wild-type mice (Csont et al., 2007). Several studies have shown that the concentration of APOB is elevated in the serum of AD patients (Caramelli et al., 1999; Sabbagh et al., 2004) which correlates with β-amyloid (Aβ) deposition in AD brains (Kuo et al., 1998). Cholesterol and apolipoprotein accumulates in mature amyloid plaques in brains from both AD patients and animal models of AD (Puglielli et al., 2003). The processing of amyloid precursor protein (APP) is modulated by cholesterol which is enriched in the membrane microdomains of neurons (Ehehalt et al., 2003). The amount of 24S-hydroxycholesterol is increased in the plasma of AD and vascular dementia patients, indicating a change in the metabolism of cholesterol (Lutjohann et al., 2000). High levels of plasma triglyceride preceded the formation of amyloid plaques in transgenic mouse models of AD (Burgess et al., 2006). In our model, APOB-100 transgenic mice showed significantly elevated serum triglyceride and cholesterol level when fed with normal chow and cholesterol rich diet, respectively (Csont et al., 2007), and increased the rigidity of the plasma membrane of brain endothelial cells isolated from these transgenic animals (Lénárt et al., 2015). In the past 10 years we have described in detail the neurodegenerative processes occurring in the brain of hypertriglyceridemic APOB-100 transgenic mice. We detected widespread neuronal cell death and apoptosis of cortical and hippocampal neurons in this model (Lénárt et al., 2012). Synaptic dysfunction in the hippocampal region of APOB-100 transgenic mice using electrophysiolology and hyperphosphorylation of the tau protein (primarily at Ser^262^, Ser^396^, Ser^199/202^, Ser^404^phosphosites) were also shown (Lénárt et al., 2012). As a consequence of the extended neurodegeneration a pronounced enlargement of brain ventricles in transgenic brains was detected using MRI, which was transgene dose-dependent (Bereczki et al., 2008). Moreover, APOB-100 overexpression increased the level of lipid peroxidation in cortical and hippocampal brain regions and impaired cognitive function of the animals (Löffler et al., 2013).

Under cerebral ischemic conditions, decreased cortical microvascular density and increased brain capillary lumen diameter was found in our APOB-100 transgenic atherosclerosis model showing neurodegeneration (Süle et al., 2009). Cerebral ischemia also promoted the swelling of perivascular astrocytes and reduced the ratio of intact capillaries (Süle et al., 2009). Our aim was to further study neurovascular pathology and reveal structural and functional changes in the BBB of APOB-100 transgenic mice that may contribute to the neurodegeneration already described in this model.

## Materials and Methods

### Animals

All animals were handled in accordance with approved procedures as defined by the EU Directive 2010/63/EU and all animal work was approved by the regional Station for Animal Health and Food Control (Csongrád-county, Hungary; project license: XVI/4136/2014). Mice were housed in groups of two to three under standard conditions (24°C, 12 h light–dark cycle) with food and water available *ad libitum*. APOB-100 transgenic mice were produced in our laboratory as described previously (Bjelik et al., 2006). Transgenic mice were backcrossed with C57B/6 strain six times to achieve a homogenous genetic background. Animals were maintained on a regular rodent chow diet. Animal surgeries were performed under sodium pentobarbital (Nembutal) anesthesia and all efforts were made to minimize pain and suffering. For genotyping, tail DNA of 10-day-old pups was purified as described earlier (Bjelik et al., 2006) and integrated transgenes were detected by PCR, using primers from the 5′ promoter region of the human *APOB* gene (Callow et al., 1994).

### Materials

All reagents were purchased from Sigma-Aldrich Ltd. (St. Louis, MO, United States) except for those specifically mentioned.

### Serum Triglyceride Measurement

Serum triglyceride levels in 7, 9, and 12-month-old APOB-100 transgenic (*n* = 5) and wild-type mice (*n* = 5) fed on a normal chow diet were measured using a colorimetric assay (Supplementary Table S1). Blood samples were collected through cardiac puncture under terminal anesthesia. After clot formation samples were centrifuged at 4°C, 1000 × *g* for 10 min, then serum was removed and stored at -80°C until use. Serum triglyceride levels were measured in triplicate using a commercially available enzymatic colorimetric assay kit (Diagnosticum Ltd., Budapest, Hungary) according to the manufacturer’s instructions. Test accuracy was monitored using Standard Lipid Controls (Diagnosticum Ltd., Budapest, Hungary). Absorbance of the produced purple color product was measured at 560 nm using a microplate reader (Multiskan FC, Thermo Scientific, United States). Values were expressed in mmol/liter. Experimental groups, (APOB-100 transgenic mice and wild-type littermates) consisted of 5 animals each.

### BBB Permeability

Permeability for sodium fluorescein (SF, mw: 376 Da), a marker of paracellular flux, and Evans blue (EB, mw: 67 kDa), a tracer which binds to serum albumin (Patterson et al., 1992), was measured as described in detail earlier (Veszelka et al., 2003). Six-month-old wild-type and transgenic mice (*n* = 10 animals/group) (Supplementary Table S1) were given a solution of both dyes (2%, 5 ml/kg) in an *iv*. injection to the tail vein for 1 h, and at the end of the experiments, the animals were perfused with 25 ml phosphate-buffered saline (PBS) for 15 min. Samples from two brain regions, cerebral cortex and hippocampus, were collected, weighed and stored at -80°C. Tissue pieces were homogenized in 650 μl PBS, then 650 μl of cold, 50% w/v, freshly prepared trichloroacetic acid was added and samples were centrifuged again with 10,000 × *g* for 12 min at 4°C. Dye concentrations were measured in supernatants by a PTI spectrofluorimeter (T-format, Quanta Master QM-1; Photon Technology International). Five hundred μl of the supernatants were diluted in ethanol (1:3) than emission of Evans blue was measured at 650 nm after excitation at 600 nm wavelength. For SF measurement 500 μl supernatants were diluted in distilled water (1:3) then 100 μl 10N NaOH was added to each sample. Emission of fluorescein was measured at 510 nm after excitation at 492 nm wavelength. BBB permeability was expressed as ng tracer/g brain tissue.

### Transmission Electron Microscopy (TEM) and Image Analysis

Seven-month-old wild-type and transgenic mice (*n* = 4 animals/group) (Supplementary Table S1) were anesthetized with sodium pentobarbital (150 μg/g, i.p.), then transcardially perfused with 0.9% NaCl in 0.01 M phosphate buffer (PB), followed by 4% paraformaldehyde containing 2.5% glutaraldehyde in 0.1 M PB. Brains were removed and post-fixed in 4% paraformaldehyde in 0.1 M PB overnight at 4°C. Then, 40-μm-thick coronal sections were cut on an Oxford Vibratome (The Vibratome Company, St. Louis, MO, United States). Sections were washed with PBS and incubated in 1% OsO_4_ for 30 min, then rinsed with distilled water and dehydrated in graded ethanol, block-stained with 1% uranyl acetate in 50% ethanol for 30 min and embedded in Taab 812 (Taab; Aldermaston, United Kingdom). Following polymerization at 60°C for 12 h, 60–70 nm ultrathin sections were cut using a Leica UCT ultramicrotome (Leica Microsystems, Milton Keynes, United Kingdom) and examined using a Hitachi 7100 transmission electron microscope (Hitachi Ltd., Tokyo, Japan). Electron micrographs were made by Veleta 2k × 2k MegaPixel side-mounted TEM CCD camera (Olympus, Tokyo, Japan). Contrast/brightness of electron micrographs was edited by Adobe Photoshop CS3 (Adobe Photoshop Inc., San Jose, CA, United States). Altogether 215 non-overlapping images representing 56 capillaries from the frontal cortex and 111 non-overlapping images representing 59 capillaries from the hippocampus were analyzed for morphological changes. All analyzed images were taken at 30,000× magnification. To calculate pericyte coverage on capillary profiles, the circumference of brain endothelial cells at their abluminal side facing the basal membrane was marked manually by a line with one color, and the length of pericytes with another. Only pericyte branches completely embedded in the capillary basement membrane were counted. The length of the lines was determined by Matlab. The background (the original image) was removed and the two colors were seperated to two channels. The pixel number of the two differently labeled lines is a good approximation of the pericyte to brain capillary endothelial circumference in each group. Thus, the ratio of the structures is the ratio of the two cumulative pixel numbers.

### Brain Microvessel Isolation

Cortical microvessels were isolated from the brain of 6–7-month-old animals, as described earlier (Veszelka et al., 2007). The forebrains of APOB-100 overexpressing or wild-type mice (*n* = 6) (Supplementary Table S1) were collected in ice-cold sterile phosphate buffered saline (PBS). Meninges were taken off by rolling brains on a sterile wet filter paper. White matter and the choroid plexus were removed and the tissue was minced into 1 mm^3^ pieces by scalpels. Samples then were homogenized in ice-cold Ringer-Hepes buffer (4 ml/g of tissue), and the resulting homogenates were centrifuged at 1000 *g* for 10 min. After centrifugation the microvessel enriched pellets were resuspended in 17.5% dextran (64–76 kDa) in Ringer-Hepes, and centrifuged at 4°C, 1500 × *g* for 15 min. The resulting pellets were suspended in 2 ml Ringer-Hepes buffer containing 1% BSA, while the supernatants were collected and centrifuged two more times. The resulting pellets were pooled and passed through a 100 μm and a 20 μm nylon mesh. The microvessels retained by the 20 μm mesh were washed off with 10 ml buffer and centrifuged at 4°C, 1000 *g* for 10 min. Finally, the pellets were resuspended in 1 ml buffer and centrifuged at 4°C, 10,000 *g* for 2 min, and stored in TRIZOL reagent until use. A small aliquot of the brain microvessel preparation was observed with phase contrast microscopy, and similarly to our previously published data (Veszelka et al., 2007), these fractions only contained brain microvessel endothelial cells and pericytes.

### Quantitative Real-Time PCR

Total RNA was extracted from primary cell cultures, cortical microvessel samples, or hippocampal and cortical brain regions using TRIZOL reagent according to the manufacturer’s protocol. Hippocampal and cortical brain samples were derived from 6-month-old mice (*n* = 6 animals/group) (Supplementary Table S1). Briefly, samples were homogenized in the appropriate volume of TRIZOL reagent, then 1/5 volume chloroform was added to each mixture and the samples were incubated on ice for 5 min. After centrifugation at 12,000 × *g* for 15 min at 4°C, the RNA containing aqueous phase was separated from the organic phase. The RNA was precipitated with 100% isopropyl alcohol and incubated for 10 min at -20°C. After centrifugation for 10 min at 12,000 × *g* at 4°C, RNA was washed with 80% ethanol and samples were centrifuged for 5 min at 12,000 × *g* at 4°C. RNA pellets were dissolved in RNase free water and then bound to RNA Clean Up column (NucleoSpin RNA clean-up kit, Macherey-Nagel) where they were treated with DNase. RNA was finally eluted from the membrane with RNase-free water, and the concentrations of the samples were measured at 230 nm using a spectrophotometer (NanoDrop ND-1000).

mRNA samples were converted to cDNA by using a reverse transcriptase kit (High Capacity cDNA Reverse Transcription Kit, Applied Biosystems). Each reaction mix consisted of 2 μg RNA (15 μl); 1.5 μl reverse transcriptase; 3 μl primer; 1.2 μl dNTP; 3 μl buffer; 6.3 μl RNase free water. The temperature profile of the reaction was the following: 10 min at 25°C, 2 h at 37°C and 5 min at 85°C (using MJ Mini - Personal Thermal Cycler, BioRad). The cDNA was finally diluted 1:20, and 9 μl of this mix was used as a template in the PCR reaction that follows. Each reaction was performed in a total volume of 20 μl containing 10 μl of 2x Power SYBR Green PCR Master Mix (Applied Biosystems), 1 μl of 5 pmol/μl primer mix (forward + reverse) and 9 μl of cDNA sample. The amplification was carried out on a RotorGene 3000 instrument (Corbett Research) with the following cycling parameters: heat activation at 95°C for 10 min; followed by 45 cycles of denaturation at 95°C for 15 s, annealing at 56°C for 15 s, and extension at 60°C for 40 s in 45 cycles. Fluorescent signals were collected after each extension step at 72°C and at the end the registration of the melting curve was performed between 50 and 95°C. The expression level of target genes was normalized to an endogenous control gene *Gapdh* or *Actb* (ΔCt). Then ΔΔCt was calculated, i.e., the relative expression of the target genes in transgenic animals was compared with the expression levels observed in wild-type animals. Fold-differences were calculated using the 2^-ΔΔCt^ formula and were expressed in percents. Gene expression changes were considered significant if the expression level dropped below 50% or showed a two-fold increase compared with control values. We studied genes involved in different molecular mechanism, such as oxidative stress, transport pathways or endothelial dysfunction. Individual genes were selected based on literature data, especially those identified in BBB transcriptome analysis in mice (Daneman et al., 2010), related to AD pathology (Wu et al., 2005), or selected for our recently published gene expression analysis of different BBB models (Veszelka et al., 2018). Primer sequences used in this study are listed in Supplementary Table S2.

### Immunohistochemistry and Confocal Microscopy

Standard immunofluorescence protocols were applied. 7–8-months-old mice (Supplementary Table S1) were terminally anesthetized with sodium pentobarbital (150 μg/g, i.p.), then transcardially perfused with 0.9% sodium chloride dissolved in 0.01 M PB, pH 7.4, followed by 3% paraformaldehyde in 0.1 M PB, pH 7.4. Brains were removed and postfixed for 4 h in the same fixative. Following fixation the brain samples were washed in 0.1 M PB, pH 7.4, and cryoprotected in 30% sucrose until saturation. Then, 30-μm-thick, hippocampus and frontal cortex containing coronal sections were cut on a cryostat (Floorstanding Cryostat MNT; Slee, Mainz, Germany), collected in 0.1 M PB, pH 7.4 containing 0.01% sodium azide (Fluka) (w/v) and were stored at 4°C.

Antigen retrieving for claudin-5 and occludin immunostainings was performed with 0.5 % Triton X-100 in PBS for 10 min, followed by an incubation in protease type XIV (1 μg/ml) dissolved in CaCl_2_ (1 mg/ml) for 7 min. Antigen retrieving for the other primary antibodies used included only Triton X-100 treatment at concentrations based on our preliminary experiments [0.1% for Pdgfrβ, 0.2% for vimentin and Gfap, 0.3% for Lox-1, and 0.5% for *P*-glycoprotein (Pgp) for details of Lox-1 immunolabeling see Supplementary Material]. Primary antibodies used were rabbit anti-claudin-5, rabbit anti-occludin (Thermo Fisher Scientific, Waltham, MA, United States), goat anti-Gfap (Abcam, Cambridge, United Kingdom), mouse anti-vimentin [Agilent (DAKO), Santa Clara, CA, United States], mouse anti-Pgp (Merck Millipore, Burlington, MA, United States), and rabbit anti Lox-1 (Abcam, Cambridge, United Kingdom), (Supplementary Table S3). Appropriate secondary antibodies conjugated with DyLight^TM^ 488 and Alexa Fluor^TM^ 594 (Jackson ImmunoResearch Europe Ltd., Cambridgeshire, United Kingdom) were applied. Sections were counterstained with DAPI, coverslipped with Confocal Matrix^®^ (Micro Tech Lab, Graz, Austria) and examined with a confocal laser scanning microscope (Olympus Fluoview FV1000, Olympus Life Science Europa GmbH, Hamburg, Germany). Images of 512 × 512 px were captured using the following microscope configuration: objective lens: UPLSAPO 60x, numeric aperture 1.35; sampling speed: 8 μs/pixel; scanning mode: sequential unidirectional. In order to obtain high resolution (1024 × 1024 px) images, Yokogawa W1/Olympus IX83-based spinning disk confocal microscope was also used for imaging Gfap, vimentin, and Pdgfrβ immunostainings using excitation and detection parameters optimized for DyLight^TM^ 488, Alexa Fluor^TM^ 594 and DAPI. 405nm (for DAPI), 488 nm (for DyLight^TM^ 488) and 561 nm (for Alexa Fluor^TM^ 594) laser excitation, and 60x objective were used for imaging. The contrast of these images showing Gfap immunostaining was increased by inverting and displaying them as gray scale images using the public domain Fiji software.

### Fluorescence Intensity Analysis

Immunostained coronal sections containing the hippocampus and cortical areas (5 animals per group, 3 sections per animal) were selected based on the principle of systematic random sampling (Gundersen and Osterby, 1981; Mayhew, 1991). Three images representing randomly selected parts of the hippocampus and frontal cortex in each section were taken with a laser scanning (for claudin-5, occludin and Pgp immunolabeling) or a spinning disk (for Gfap immunostaining) confocal microscope using a 60× objective lens and the same excitation and detection parameters for each image. Fluorescence intensity of claudin-5, occludin and Pgp immunolabelings was evaluated using the ImageQuant^TM^ software as follows: on every image 10 equally sized small rectangular areas (7 × 7 pixels) were placed randomly on the intensively highlighted immunolabeled structures, and five equal rectangles were placed randomly on areas lacking immunostaining representing the background. Then the average intensity/pixel values of each area were calculated, and the average intensity/pixel values representing the background intensity were subtracted from those of immunolabeled areas. The GFAP immunostained structures represented a more a complex pattern and occupied larger areas, therefore the integrated fluorescence intensity of Gfap immunolabeling was measured using the public domain Fiji software as follows: Grayscale 16-bit images were used. The integrated fluorescence intensity of the whole image and that of a small area lacking immunostained structures were measured, then the background fluorescence intensity of the whole image was calculated and subtracted from the integrated fluorescence intensity value of the whole image. The procedures were performed on each image, and the collected data were statistically analyzed (Farkas et al., 2008).

### Statistical Analysis

GraphPad Prism 5.0 software (GraphPad Software Inc. LaJolla, CA, United States) was used for statistical analysis. Gaussian distribution of the data was tested with the Kolmogorov–Smirnov normality test. Data showing Gaussian distribution were analyzed with two-way analysis of variance followed by Bonferroni *post hoc* test. Data showing no Gaussian distribution were analyzed with Kruskal–Wallis and Dunn’s multiple comparison tests. The level of statistical significance was taken as *p* < 0.05. Results are presented as means ± SEM.

## Results

### Chronic Hypertriglyceridemia in APOB-100 Transgenic Mice

**FIGURE 1:**
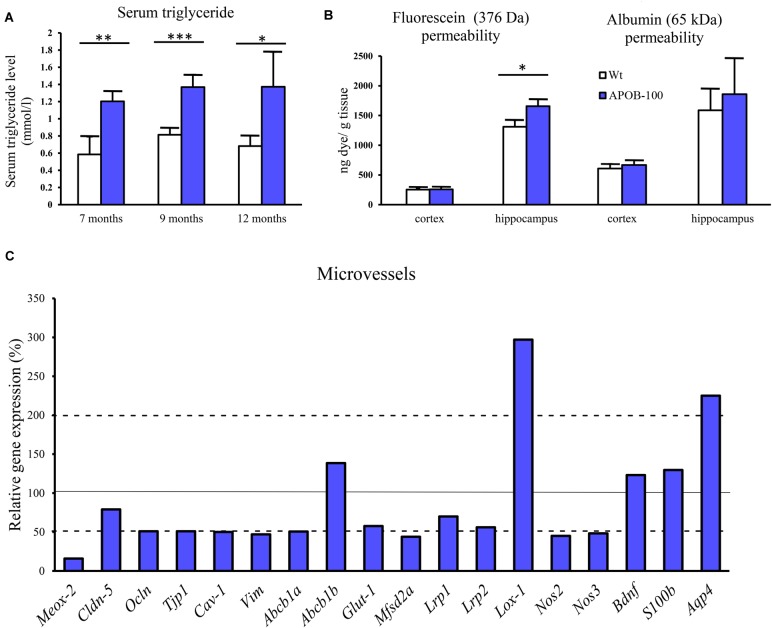
Serum triglyceride levels **(A)**, BBB permeability for fluorescein and albumin in the brain of hypertriglyceridemic APOB-100 transgenic (APOB-100) and wild-type (Wt) mice **(B)**, and gene expression analysis of the microvessel fraction from APOB-100 transgenic mice using QPCR **(C)**. Continuous line indicates the expression level of the corresponding gene in wild-type mice (100%). Dashed lines indicate the levels of significant changes: a 2 fold (200%) increase or an 0.5 fold (50%) reduction in gene expression. ^∗∗∗^*P* < 0.001, ^∗∗^*P* < 0.01, ^∗^*P* < 0.05, compared with Wt mice.

A statistically significant difference was detected in transgenic compared to wild-type animals at every time point (7, 9, and 12-month, *n* = 5 animals/group) indicating chronic hypertriglyceridemia in APOB-100 transgenic mice (Figure 1A).

### Impairment of the BBB Integrity: Permeability Measurements

We measured a significant increase in the BBB permeability (*p* < 0.05) for the small molecular weight marker SF in the hippocampal region of transgenic mice, while alteration in the extravasation of the large serum protein albumin showed a non-significant trend (Figure 1B). However, there was no obvious change in the permeability for either of the markers in the cortex of transgenic mice compared to wild-type littermates.

### BBB Dysfunction: Changes in Gene and Protein Expression in Brain Microvessels

Reduced expression (16%) of the vascular restricted and mesenchyme homeobox gene 2 (*Meox2*), a key regulator of BBB functions, particularly in AD, was measured in cerebral microvessels of APOB-100 transgenic mice compared to wild-type animals (Figure 1C). The gene expression of *Mfsd2a* (44%), the BBB transporter for unsaturated lipid docosahexaenoic acid (DHA), and the primary glucose transporter in brain endothelial cells, *Glut-1* (57%) were also decreased (Figure 1C).

Lox-1, a lectin-like protein expressed in endothelial cells in the periphery is considered as the major receptor for oxidized low-density lipoprotein. The level of oxidized LDL (oxLDL) receptor (*Lox-1*) has increased dramatically (297%) in the microvessels of APOB-100 transgenic mice (Figure 1C). In contrast to the periphery, where Lox-1 was detected in heart coronal vessels, Lox-1 immunolabeling was observed only in neuronal cell bodies and processes, but not in brain capillaries (Supplementary Figure S1). A significant increase in Lox-1 immunoreactive area was detected in the cortex, but no change was seen in the hippocampus of transgenic mice compared to wild-type animals (Supplementary Figure S2). While mRNA level of LDL receptor-related protein-1 (*Lrp1*, 70%) slightly decreased, the expression level of *Lrp2* dropped to 56% in transgenic microvessels (Figure 1C).

We measured two isoforms of nitric oxide (NO) synthases (NOS), the endothelial (*eNos/Nos3*) and the inducible form (*iNos/Nos2*), and found a significant decrease for both genes in brain microvessels of transgenic animals (Figure 1C). Expression of mRNA for caveolin-1 (*Cav-1*), a structural element of brain endothelial caveolae associated with Nos3, was also reduced (Figure 1C).

**FIGURE 2:**
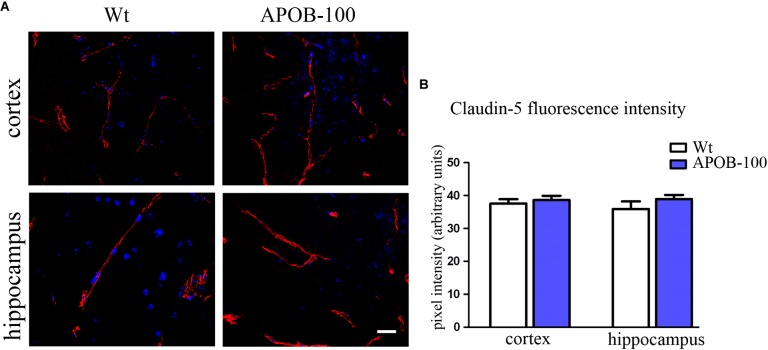
Claudin-5 immunostaining pattern (red) in the cortex and hippocampus of wild-type (Wt) and APOB-100 transgenic mice (APOB-100), counterstained with DAPI (blue) **(A)**. Scale bar: 20 μm. Quantification of fluorescence intensity of claudin-5 immunolabeling in the frontal cortex and hippocampus of Wt and APOB-100 transgenic mice **(B)**.

**FIGURE 3:**
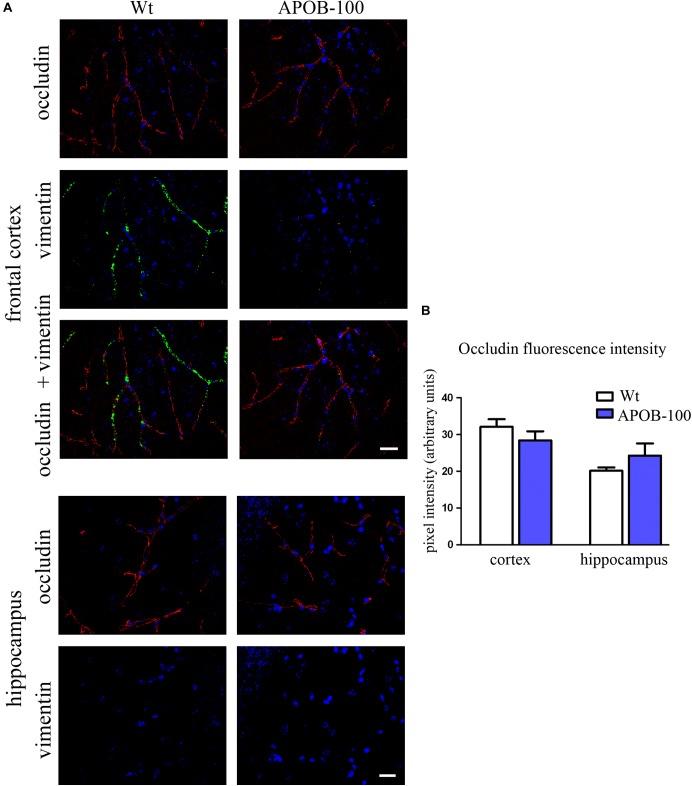
Occludin (red) and vimentin (green) immunostaining pattern in the cortex and hippocampus of wild-type (Wt) and APOB-100 transgenic mice (APOB-100) counterstained with DAPI (blue) **(A)**. Scale bar: 20 μm. Quantification of fluorescence intensity of occludin immunolabeling in the frontal cortex and hippocampus of Wt and APOB-100 transgenic mice **(B)**.

The expression of selected TJ proteins was analyzed at gene expressional level using quantitative real-time PCR, and at protein level using fluorescence immunohistochemistry, too. The mRNA level of transmembrane protein *Ocln*, and TJ cytoplasmic linker *Tjp-1*, was reduced to half in transgenic microvessels, while there was no change in the expression level of *Cldn-5*, the dominant claudin member at the BBB, compared to wild-type animals (100%) (Figure 1C). Fluorescence immunohistochemical stainings for claudin-5 (Figure 2A) and occludin (Figure 3A) showed that both immunolabelings were exclusively localized at endothelial TJs and appeared as continuous lines. There was no statistically significant change neither in claudin-5 nor in occludin immunostaining intensity in transgenic cortex and hippocampus compared to wild-type animals (Figures 2B, 3B).

**FIGURE 4:**
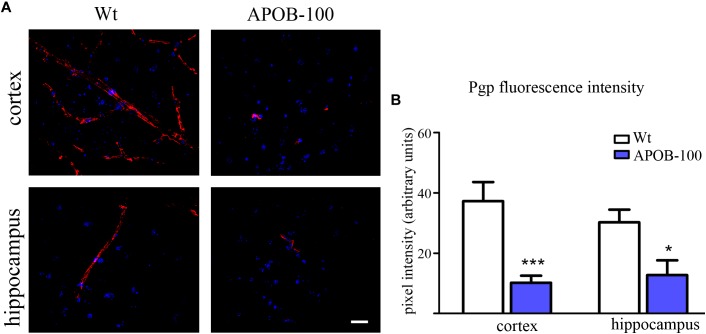
P-gp (ABCB1) transporter immunostaining pattern (red) in cortical and hippocampal areas of wild-type (Wt) and APOB-100 transgenic mice (APOB-100), counterstained with DAPI (blue) **(A)**. Scale bar: 20 μm. Quantification of fluorescence intensity of P-gp immunolabeling in the frontal cortex and hippocampus of Wt and APOB-100 transgenic mice **(B)** ^∗∗∗^*P* < 0.001, ^∗^P < 0.05, compared with Wt mice.

The gene expression of vimentin, a cytoskeletal protein, which labels reactive astrocytes (Pekny et al., 2007), but can be produced by pericytes (Bandopadhyay et al., 2001) and brain endothelial cells (Deracinois et al., 2013) too, showed a significant reduction in isolated brain microvessels (Figure 1C). At protein level, vimentin was localized along capillaries in the frontal cortex of wild-type mice as it can be observed in occludin-vimentin double immunofluorescence stainings (Figure 3A), where occludin immunolabeling delineates brain capillaries. Vimentin immunoreactivity was very rarely seen in the frontal cortex of APOB-100 transgenic animals, and it was not detected in the hippocampus of either genotype (Figure 3A). The vimentin immunolabeling showed no co-localization with epitopes recognized either by a Gfap antibody (an astroglia marker), or by a Pdgfrβ antibody (a pericyte marker) (Supplementary Figure S3). Furthermore, we have established primary pericyte, astrocyte and endothelial cell cultures from wild-type and transgenic mice and vimentin gene expression was measured using QPCR. The vimentin gene expressions normalized to the endogenous mouse actin gene were compared in wild-type animals (ΔCT values). Vimentin was expressed at high level in endothelial cells (ΔCT = 1.3), astrocytes (ΔCT = 2) and pericytes (ΔCT = 2.3) as well. Comparison of the vimentin expression level of wild-type and transgenic animals demonstrates that while the vimentin expression levels were not changed significantly in the transgenic endothelial cells and astrocytes (74 and 124%, respectively) transgenic pericytes showed significantly reduced vimentin level (33%) compared to wild-type cells (Supplementary Figure S4). The most widely studied, and AD related ABC transporter at the BBB, *Abcb1* or P-glycoprotein (P-gp) was also examined by QPCR in isolated brain microvessels and by immunohistochemistry in the frontal cortex and hippocampus of wild-type and APOB-100 transgenic mice. From the isoforms of P-gp coding genes, *Abcb1a* mRNA expression was significantly decreased (51%), while *Abcb1b* (138%) showed no significant change (Figure 1C). Using a monoclonal antibody recognizing both isoforms of P-gp the immunoreactivity pattern of P-gp was similar to that of TJ proteins delineating a large number of capillaries in the frontal cortex and hippocampus in wild-type animals. However, in the APOB-100 transgenic group, P-gp immunoreactivity could hardly be observed in either brain region examined (Figure 4A), which was reflected by a significant drop in P-gp fluorescence intensity in APOB-100 transgenic mice compared to wild-type animals in the cortex and hippocampus, too (Figure 4B).

Astroglial endfeet are important structural and functional elements of the BBB. The gene expression level of aquaporin-4 (*Aqp4*), a water channel and a marker of glial endfeet, has increased dramatically (225%) in isolated brain microvessels (Figure 1C), while no change was observed in brain samples (Figure 6). In the microvessel samples the glial specific calcium-binding cytoplasmic protein *S100b* (129%) and the astrocyte produced brain derived neurotrophic factor (*Bdnf*, 123%) levels showed no change (Figure 1C).

### Ultrastructural Changes of the BBB in APOB-100 Transgenic Mice

**FIGURE 5:**
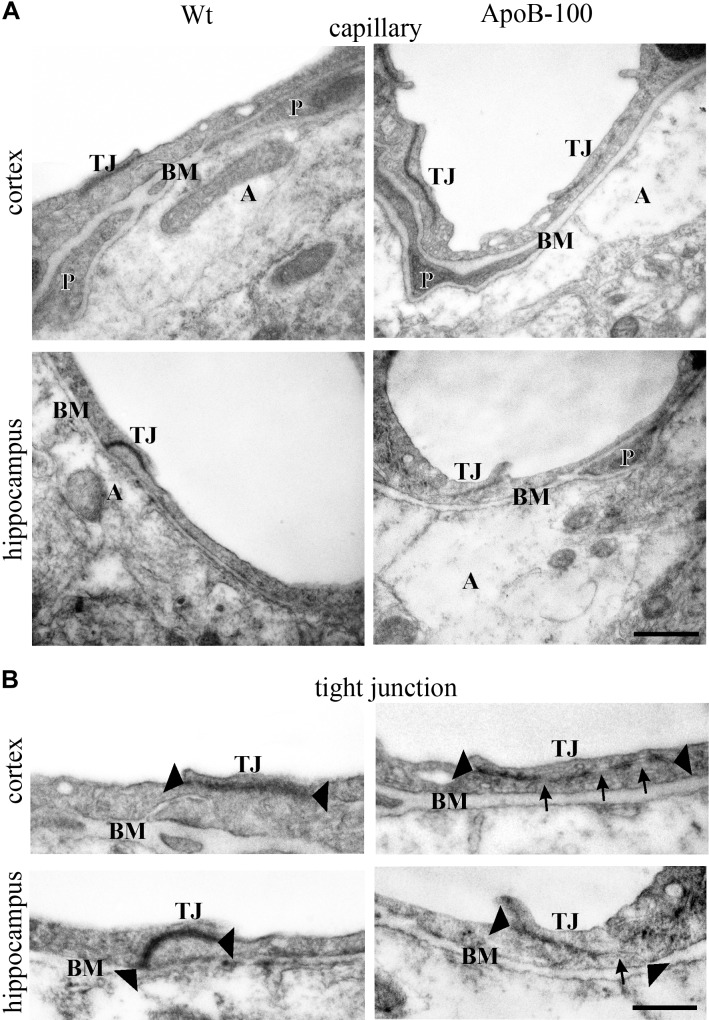
Electron micrographs of brain capillaries **(A)** and tight junctions **(B)** in the frontal cortex and hippocampus of wild-type (Wt) and APOB-100 transgenic (APOB-100) mice. Tight junctions in **(B)** are larger magnifications of tight junctions seen in **(A)**. A, astrocyte; BM, basal membrane; P, pericyte; TJ, tight junction. Scale bars: 500 nm **(A)** and 800 nm **(B)**.

Analysis of the ultrastructure of brain capillaries using TEM revealed several alterations in the NVU of APOB-100 transgenic mice compared to wild-type animals (Figure 5 and Table 1). The most significant changes were observed in the morphology of TJs and astrocytic endfeet. Swollen astrocytic processes around capillaries were predominant in the transgenic group, indicating edema of glial endfeet (Figure 5A and Supplementary Figure S5). The intercellular junctions of capillary endothelial cells were characterized by a continuous electron dense material in wild-type mice (Figure 5B). In the transgenic group, in contrast, nearly half of the endothelial cell contacts displayed a discontinuous electron dense structure (Figure 5B and Table 1). Further ultrastructural differences include basal membrane alterations, like increased thickness, tortuosity, fragmentation, and alterations in luminal membrane characteristics, like increased number of protrusions in the transgenic group. No significant difference was found in the pericyte coverage of brain capillaries (Table 1). The described ultrastructural changes were detected in the frontal cortex and in the hippocampus as well.

**Table 1:** Summary of changes in BBB ultrastructure in the frontal cortex and hippocampus of wild-type (Wt) and APOB-100 transgenic (APOB-100) mice.

Frontal cortex		Wt	APOB-100
Number of capillaries		23	33
Number of images		91	124
**Capillary endothelial cell**			
Luminal membrane	Smooth	69%	48%
	Protrusions	31%	52%
Tight junctions	Intact	100%	56%
	Discontinuous	0%	44%
Basal membrane	Intact	78%	56%
	Altered	22%	44%
Pericyte			
	Capillary coverage	20%	17%
	Capillary wo pericyte	5%	22%
Astrocyte			
	Intact	77%	12%
	Edema	23%	88%

**Hippocampus CA1**		**Wt**	**APOB-100**

Number of capillaries		29	30
Number of images		92	129
**Capillary endothelial cell**			
Luminal membrane	Smooth	78%	46%
	Protrusions	22%	54%
Tight junctions	Intact	100%	29%
	Discontinuous	0%	71%
Basal membrane	Intact	89%	24%
	Altered	11%	76%
Pericyte			
	Capillary coverage	25%	23%
	Capillary wo pericyte	19%	23%
Astrocyte			
	Intact	89%	44%
	Edema	11%	56%


*Values are shown as percent of all images analyzed in the group. Pericyte coverage was calculated as written in the Materials and methods section.*

### Neuroinflammation and Neuron Related Changes in the Cortex and Hippocampus

**FIGURE 6:**
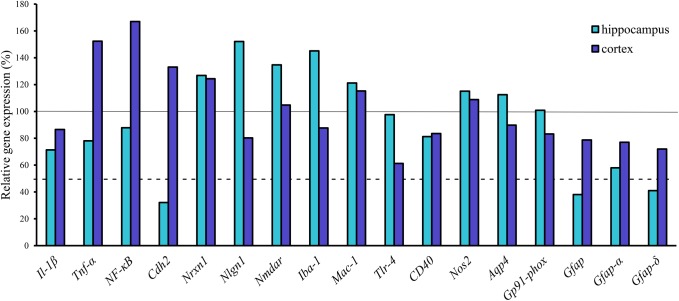
Gene expression analysis of neuroinflammatory markers, transporters and Gfap isoforms in the hippocampus and cortex of APOB-100 transgenic mice. Continuous line indicates the expression level of the corresponding gene in wild-type mice (100%). Dashed line indicates the level of significant changes: a 0.5 fold (50%) reduction in gene expression.

From the genes of selected cytokines and neuroinflammatory markers the expression of cytokine *Tnf*α (152%) and the inflammation related nuclear factor *NF-κB* (167%) increased in the transgenic cortex but were not substantially changed in the hippocampus. No change was measured for *Il-1*β (Figure 6). Similarly, there was no notable change in the expression of other inflammatory markers in the cortex, except for *Tlr4*, the expression of which was dropped down to 60%. Of neuronal synaptic genes, which play an important role in remodeling CNS synapsis, the level of *Nlgn1* (152%) has slightly increased in the hippocampus but was not changed in the cortex. The expression level of *Nmdar* and *Nrxn1* was unchanged both in the hippocampus and in the cortex. However, a significant decrease in the expression level of a cell adhesion molecule, *Cdh2* (*N-cadherin)*, (32%) was detected in the hippocampus of transgenic brains (Figure 6). The results of gene expression analysis are summarized in Table 2.

**Table 2:** Summary of gene expression changes in hypertriglyceridemic APOB-100 transgenic mice.

	mRNA		Protein	
	
	Tissue	Level	Tissue	Level
P-gp (Abcb1a)	Microvessel	Decreased	Hippocampus	Decreased
			Cortex	Decreased
iNOS	Microvessel	Decreased		n.d.
eNOS	Microvessel	Decreased		n.d.
Lox1	Microvessel	Increased	Cortex	Increased
Aqp4	Microvessel	Increased		n.d
Occludin	Microvessel	Decreased	Hippocampus	No change
			Cortex	No change
ZO-1	Microvessel	Decreased		n.d
Caveolin-1	Microvessel	Decreased		n.d
Vimentin	Microvessel	Decreased	Cortex	Decreased
Mfsd2A	Microvessel	Decreased		n.d
Meox-2	Microvessel	Decreased		n.d.
Glut-1	Microvessel	Decreased		n.d
Bdnf	Cortex	Increased		n.d.
Aif-1/Iba-1	Hippocampus	Increased^∗^	Hippocampus	No change
Gfap	Hippocampus	Decreased	Hippocampus	No change
			Cortex	Decreased
Gfap alpha	Hippocampus	Decreased		n.d
Gfap delta	Hippocampus	Decreased		n.d.
IL-β	Hippocampus	Decreased^∗^		n.d
Ncadh	Hippocampus	Decreased		n.d.
TNF-α	Cortex	Increased^∗^		n.d
NF-κB	Cortex	Increased^∗^		n.d.
Tlr4	Cortex	Decreased^∗^		n.d


*^∗^Denotes less than two-fold change.*

Out of 600 miRNAs analyzed, we could detect 10 (1.67%) differentially expressed miRNA genes in APOB100 transgenic cortices (6 upregulated and 4 repressed). List of miRNA genes with altered expression level is shown in Supplementary Table S4. Overexpressed miRNAs were mmu-miR-669g, mmu-miR-222, mmu-miR-708, mmu-miR-26a, mmu-miR-1898, and mmu-miR-500, and repressed miRNAs were mmu-miR-7a, mmu-miR-7b, mmu-miR-187, and mmu-miR-1a. Although we found 10 miRNAs showing differential expression, only 4 miRNAs had experimentally validated targets in the IPA database (mmu-miR-1a, mmu-miR-222, mmu-miR-26a, mmu-miR-7b). Some of these target genes (*n* = 11) were expected to have relevance in neuronal changes. The expression level of these genes was further investigated by QPCR but no significant changes were detected (data not shown) with the exception of *Bdnf*. This gene may be regulated by two differentially expressed miRNAs: mmu-miR-26a (overexpressed), and mmu-miR-1a (repressed). QPCR data showed a two-fold overexpression of *Bdnf* gene in the transgenic cortex (Supplementary Figure S6).

### Changes in Glial Cell Gene Expression and Morphology

**FIGURE 7:**
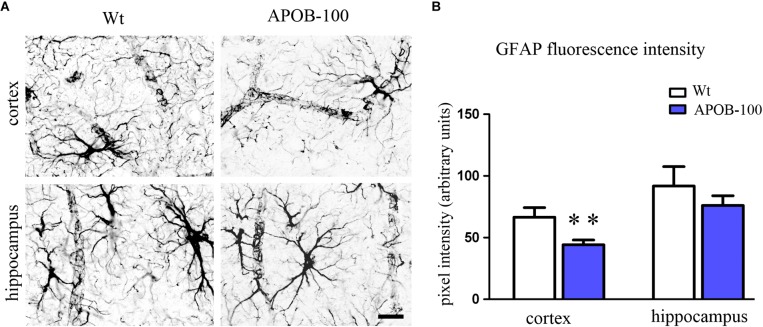
Gfap immunostaining pattern in the cortex and hippocampus of wild-type (Wt) and APOB-100 transgenic mice (APOB-100) **(A).** Scale bar: 25 μm. Quantification of fluorescence intensity of Gfap immunolabeling in the frontal cortex and hippocampus of Wt and APOB-100 transgenic mice **(B)**. ^∗∗^*P* < 0.01 compared with Wt mice.

The expression level of the microglial marker (*Iba-1/Aif1*) was slightly increased (145%) in the hippocampus of APOB-100 transgenic mice compared to wild-type animals (Figure 6). In contrast, the expression level of glial fibrillary acidic protein (*Gfap*), a cytoskeletal astroglial marker, was dropped down to 38% in transgenic hippocampus (Figure 6). The level of two *Gfap* isoforms, *Gfap*-α and *Gfap*-σ, was also reduced to 59 and 41%, respectively, in the hippocampus of APOB-100 transgenic mice compared to wild-type littermates (100%) (Figure 6). Gfap immunoreactive cells displayed several processes that were closely associated with microvessels in the cortex and in the hippocampus too, in both experimental groups (Figure 7A). The intensity of the fluorescent Gfap immunolabeling showed a significant decrease in the cortex of APOB-100 transgenic mice compared to wild-type animals (Figure 7B).

## Discussion

Here, we describe, that transgenic mice overexpressing the human APOB-100 protein show chronic hypertriglyceridemia, an increase in permeability for a small molecular marker, and gene expressional, immunohistochemical and ultrastructural alterations at the BBB. Several previous reports have already shown that hypertriglyceridemia is a serious risk factor in the development of neurodegeneration and dementia (Burgess et al., 2006; Raffaitin et al., 2009; Bowman et al., 2012; Klafke et al., 2015) and it was also demonstrated that dyslipidemia is more prevalent in AD subjects with BBB impairment (Bowman et al., 2012). Observations in aging rats, that a decrease in cerebral blood flow is linked to pathologies similar to those found in AD (De La Torre et al., 1992; De la Torre and Mussivand, 1993) and demonstration of brain microvascular injury and BBB leakage in AD patients (Zipser et al., 2007) led to the vascular concept of AD which became generally accepted by today (Zlokovic, 2008; Gosselet et al., 2013; Zhao et al., 2015; Kisler et al., 2017). Lipolysis products generated from triglyceride rich lipoproteins damage endothelial barrier function: perturb the expression of junctional proteins, induce apoptotic cell death *in vitro* in human aortic endothelial cells (Eiselein et al., 2007) and transiently elevate BBB permeability *in vivo* in mice (Lee et al., 2017). Hypertriglyceridemia may contribute to endothelial dysfunction likely through the generation of oxidative stress (Antonios et al., 2008). Indeed, in our recent study (Lénárt et al., 2015) we have shown that oxidized LDL treatment induced barrier dysfunction and increased reactive oxygen species production and membrane rigidity in primary brain endothelial cells.

In the present paper we studied BBB related functional and morphological characteristics as well as gene expression profiling of APOB-100 transgenic mice. We found APOB-100 transgenic mice are characterized by chronic hypertriglyceridemia. Regarding BBB function, a significantly increased extravasation for a small molecule marker, SF, was observed in the hippocampus of transgenic mice, which may suggest an increase in paracellular permeability. Gene expression changes suggesting endothelial dysfunction in APOB-100 transgenic animals include reduced expression of the homeobox regulator *Meox2*, and BBB transporters *Mfsd2a*, *Glut1, Lrp2*, *Abcb1a* genes in APOB-100 transgenic brain microvessels may indicate pathomechanisms similar to those observed in conditions of BBB dysfunctions (Zlokovic, 2011; Zhao et al., 2015). Low expression of *MEOX2* was demonstrated in cultured brain endothelial cells isolated from severely affected AD patients (Wu et al., 2005). In the same study Meox2 deletion in mice resulted in decreased density of brain capillaries, lower levels of cerebral blood flow during rest, a diminished hypoxia-induced angiogenic response in the brain. In addition, in these animals low levels of LRP were observed leading to reduced Aβ efflux. Our observations on the drastically reduced *Meox2* in transgenic brain microvessels might also support the link between Meox2 and neurovascular dysfunction. Beside *Meox2* the other gene for which a dramatic decrease was observed in brain microvessels from transgenic animals was *Mfsd2a*, which might have contributed to the increased BBB permeability in our study and the neurodegeneration in our model described previously (Bereczki et al., 2008). It has been recently discovered, that Mfsd2a is not only a DHA transporter at the BBB, but also a key regulator of BBB integrity and function (Zhao and Zlokovic, 2014). Mutations in *MFSD2A* gene leads to decreased DHA transport into the CNS and severe neurological symptoms (Betsholtz, 2015), and DHA can protect cells of the NVU against amyloid peptide toxicity (Veszelka et al., 2013). At the BBB GLUT1 is the main transporter of glucose, the primary energy source in the CNS. A reduction in Glut1 expression was linked to neurovascular dysfunction and AD in a mouse model (Winkler et al., 2015) emphasizing the importance of the present findings.

Additionally, the receptors Lrp1, Lrp2 and the transporter P-gp responsible for the clearance of Aβ from the brain (Shibata et al., 2000; Deane et al., 2003; Cirrito et al., 2005) are downregulated in AD pathology (Marques et al., 2013). In this study we showed a significant reduction in the expression of the *Abcb1a* gene coding the P-gp isoform predominantly expressed in brain capillary endothelial cells (Croop et al., 1989; Shoshani et al., 1998). In agreement with this finding, the P-gp immunolabeling could hardly be detected in APOB-100 transgenic mouse brains, which may be linked to the previously demonstrated increased accumulation of Aβ in this model (Bereczki et al., 2008).

A significant decrease in inducible *Nos2* and endothelial *Nos3*, and an increase in *Lox-1* expression were detected in microvessels isolated from APOB-100 transgenic animals. The area fraction of Lox-1 immunoreactive structures was also significantly increased in the cortex of APOB-100 transgenic mice compared to wild-type animals. The upregulation of *Lox-1* and the downregulation of *Nos2* and *Nos3* mRNA levels confirmed atherosclerotic changes in APOB-100 transgenic mice reported earlier (Chen et al., 2002; Csont et al., 2007) The reduction in *Nos3* gene expression is in line with observations focusing on atherosclerosis-prone regions of the mouse aorta (Won et al., 2007) and on areas of human atherosclerosis (Buttery et al., 1996; Barry et al., 1998). Lox-1 upregulation is observed in atherosclerosis and linked to oxidative stress and inflammatory reactions leading to inhibition of Nos3 enzymatic activity via increased C-reactive protein production (Stapleton et al., 2010; Stancel et al., 2016) and, consequently, microvessel dysfunction in the periphery (Lubrano and Balzan, 2016). Our study suggests for the first time, that there may be a link between neurovascular changes and *Lox-1* upregulation, *Nos2* and *Nos3* downregulation in brain microvessels.

NO or NO donors at low concentrations do not modify the barrier function of BBB culture models, while both the blocking of basal NO production and high levels of NO induce barrier opening (Deli et al., 2005). NO is known to modulate cGMP-pathways which decrease resistance and increase permeability in culture models of the BBB and may mediate the effects of excess NO (Deli et al., 2005), but the exact mechanisms by which NO regulates different BBB permeability pathways are not known.

A significant decrease in *Cav-1* gene expression was detected in microvessels isolated from APOB-100 transgenic brains. Caveolin-1 is a structural protein playing a stabilizing role in caveolae, but it is reported to influence TJ morphology and the expression of junctional proteins occludin and ZO-1, too. Caveolin-1 knock-out mice are characterized by increased paracellular permeability, smaller TJs and defects in the adhesion of endothelial cells to the-basement membrane (Schubert et al., 2002). Knocking down caveolin-1 expression in brain endothelial cells resulted in a decrease in occludin and ZO-1 expression in western blots (Song et al., 2007). However, the effect of caveolin-1 on TJ protein expression is contradictory, since an increase in caveolin-1 expression was also linked to a decrease in occludin and or claudin-5 protein expression related to BBB damage (Nag et al., 2007; Beauchesne et al., 2010). In our study a decrease was observed in both caveolin-1 and occludin gene expression using QPCR, which is in concordance with the western blot findings reported by Song et al. (2007).

The increase in BBB permeability in our model may be linked to reduced expression of genes coding important brain endothelial TJ proteins *Cldn-5, Ocln, Tjp-1*. However, the differences detected at mRNA level were not reflected by the immunofluorescent staining pattern of TJ proteins claudin-5 and occludin, suggesting no detectable changes of TJs between the experimental groups at light microscopic level. In contrast, regarding the ultrastructure, a great percentage of discontinuous TJs were observed in TEM images of transgenic brains. Data obtained by PCR and immunohistochemistry do not necessarily show direct positive correlation due to the complex regulation of the gene expression at transcriptional, posttranscriptional, and posttranslational levels resulting in divergences (Vogel and Marcotte, 2012). In addition, during tissue fixation some epitopes may suffer alterations resulting in changes in specific antibody binding. Consequently, both these possibilities may explain the differences between PCR data and the analysis of immunofluorescent TJ signals.

Our findings on functional and gene expressional alterations at the BBB in APOB-100 mice are in agreement with the neurovascular concept of AD. Barrier dysfunction in brain microvessels is well described in AD (Zipser et al., 2007; Nelson et al., 2016), and BBB breakdown was found in the hippocampus in aging human brain which may contribute to cognitive impairment (Montagne et al., 2015).

Due to the observed similarities in gene expression changes present in both AD pathology and in APOB-100 transgenic mice, we examined whether neuroinflammation and neuronal changes were also characteristic features of our model. An increase in the gene expression of *Tnf*α, *NF-κB* and *Aif-1*/*Iba-1* detected in the brain of APOB-100 transgenic mice suggested inflammation related changes. Among the other inflammatory markers examined, *Tlr4*, which is reported to participate in innate neuroprotective mechanisms (Conte et al., 2017), decreased to 60% in the cortex of APOB-100 transgenic mice. It may indicate a disturbance in neuroprotection. Furthermore, a significant decrease in *Cdh2* expression coding *N*-cadherin was observed in the hippocampus of APOB-100 transgenic animals. *N*-cadherin, as an adhesion molecule, plays an important role in connecting pericytes to endothelial cells, thus maintaining normal BBB integrity (Winkler et al., 2011). Consequently, a loss in *N*-cadherin might lead to the structural and functional disintegration of the BBB.

The miRNA analysis revealed 10 miRNAs showing altered expression in APOB-100 transgenic cortex compared with wild-type ones. Out of these miRNAs mmu-miR-1a, mmu-miR-222, mmu-miR-26a, mmu-miR-7b are correlated with validated target genes. Among the target genes only *Bdnf* can be related to neuronal changes. It is regulated by several miRNAs (Varendi et al., 2014), among others mir-1, miRNA-26a and 26b suppress endogenous BDNF protein levels (Caputo et al., 2011; Varendi et al., 2014) In our study, we measured elevated *Bdnf* mRNA level, while the expression level of two posttranscriptional regulator miRNAs was changed in the opposite direction, miR-1a was downregulated and inversely, miR-26b was upregulated. The reduced expression of miR-1a and the increased expression of miR-26a is in accordance with findings related to high cholesterol levels and AD, respectively. High-cholesterol diet induced a significant decrease of miR-1 expression in ApoE deficient mice (Wang et al., 2013). Blocking miR-1 with antagomir enhanced endothelial permeability, while miR-1 mimics attenuated endothelial barrier dysfunction, strongly indicating that miR-1 contributes to the regulation of endothelial barrier function (Wang et al., 2013). Similarly to our result, miR-26a was shown to be upregulated in AD brains (Cogswell et al., 2008) indicating that miR-26a might serve as a therapeutic target for patients with AD (Li and Sun, 2011).

In addition to brain capillary endothelial cells and neurons, alterations in other structural components of the NVU, such as astrocyte endfeet and pericytes may also contribute to BBB dysfunction. Astrocytes participate in the development and maintenance of BBB features, including tightening of TJs, inducing the expression of influx and efflux transporters, as well as specialized enzyme systems (Deli et al., 2005; Abbott et al., 2006; Deracinois et al., 2013). The expression level of Gfap, an astroglia marker, is critical in maintaining BBB integrity. Either an increase or a decrease in Gfap expression results in TJ protein expressional changes and BBB dysfunction (Willis, 2012). The fluorescence intensity of Gfap immunolabeling was significantly decreased in the cortex of APOB-100 transgenic mice compared to their wild-type littermates, which may contribute to the observed decrease in the expression of various TJ protein coding genes. It also may be in line with ultrastructural changes of astrocytes, characterized by edematic swellings around capillaries in the brain of APOB-100 transgenic mice. In a recent study (Ito et al., 2017), an increased sodium fluorescein permeability was reported following electroconvulsive stimulations. Regarding brain capillary ultrastructure, intact TJs were detected in this model of epilepsy, while astrocytic endfeet were swollen. In another paper (Haley and Lawrence, 2017) increased BBB permeability and astrocytic endfeet swelling occurred after cerebral ischaemia, but TJs remained intact and TJ proteins claudin-5 and occludin expression showed no change in western blots. Our study is in concordance with these data on both astroglia morphology and TJ protein expression. The abnormalities in astrocytic endfeet may be linked to the increased expression of *Aqp4* in brain microvessels isolated from APOB-100 transgenic animals. The loss of polarized expression of AQP4 in astrocyte foot processes has an important impact on development of BBB dysfunction and perivascular edema, and also disturbed homeostasis in the brain parenchyma in various pathologies (Wolburg et al., 2009).

S100b, another predominantly glial protein may also indicate BBB disruption. S100B is considered a plasma biomarker in traumatic brain injury (Halstrom et al., 2017), and cerebral small vessel disease, in which it is associated with cognitive impairment in patients (Gao et al., 2015). S100B is also linked to pathological changes observed in early AD (Mattsson et al., 2014). In our model, no significant difference was found in *S100b* gene expression in the transgenic microvessels. In contrast, the gene expression of vimentin, another intermediate filament protein, dropped to less than 50% in the microvessel fraction of APOB-100 transgenic mice. The decrease in vimentin expression was observed at protein level too, based on immunohistochemical findings. Vimentin is expressed by endothelial cells, fibroblasts and α-smooth muscle actin producing cells in the middle cerebral artery of hyperlipidemic rabbits (Kacem et al., 2006). In an attempt to identify which cell type of the NVU is producing vimentin in our experimental animals, double immunolabeling studies were performed. Vimentin did not colocalize either with Gfap or Pdgfrβ, but a drop of vimentin mRNA expression was only observed in cultured pericytes from transgenic mice. Although cultures do not fully reflect the *in vivo* situation, the immunohistochemistry data together with the vimentin QPCR results on microvessels and cultured cells point to the possibility, that the change in the vimentin staining in brain microvessels of APOB-100 transgenic mice is related to pericytes. The reduction in vimentin immunostaining in the cortical microvessels of APOB-100 transgenic mice reflects that vimentin expression may react very sensitively to hypertriglyceridemic conditions.

Pericytes are embedded between endothelial cells and astrocyte endfeet in brain capillaries and they are crucial components of the NVU. They induce the expression of BBB-specific genes in cerebral endothelial cells and the polarization of glial endfeet which cover brain microvessels (Armulik et al., 2010). Pericyte deficiency leads to accelerated amyloid angiopathy and cerebral β-amyloidosis in a mouse AD model (Sagare et al., 2013). In our TEM analysis we detected an increase in the ratio of capillaries displaying no pericyte branches in the frontal cortex of APOB-100 transgenic mice compared with wild-type animals. It may suggest a decrease in pericyte number, which is in accordance with earlier findings demonstrating a reduction in the number of pericytes during neurodegeneration (Sagare et al., 2013).

## Conclusion

**FIGURE 8:**
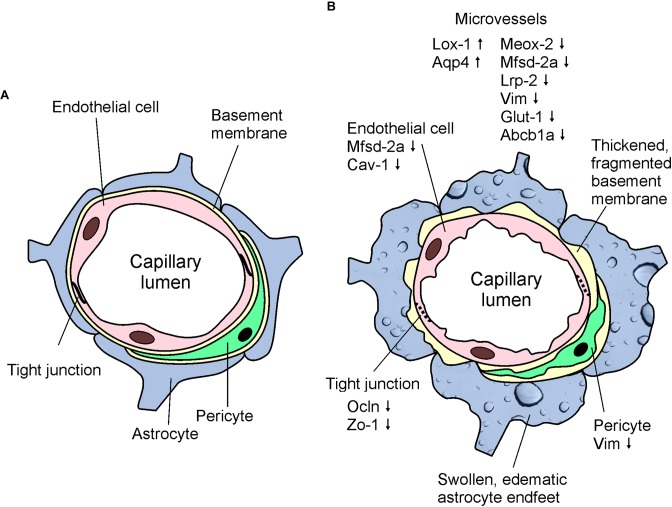
Schematic drawings showing the basic structure of the BBB **(A)** and highlighting the most characteristic morphological and gene expression changes detected in cerebral microvessels of APOB-100 transgenic mice **(B)**.

In conclusion, we demonstrated here, that APOB-100 transgenic mice are characterized by elevated serum triglyceride levels and show functional, morphological, and gene expression alterations suggesting BBB dysfunction (Figure 8). Based on our findings we propose APOB-100 transgenic mice as a novel mouse model of vascular neurodegeneration. This model might provide researchers a useful tool to gain deeper insights into the pathomechanism of neurodegenerative diseases of vascular origin, which is fundamental for the development of efficient therapies.

## Author Contributions

MD, ZH, MT, and MS contributed to the conception and design of the study. DN, NL, and MT performed the BBB penetration study. ÁK, ZH, MT, and MD conducted the ultrastructural studies. ZH, MT, BD, FW, SV, JV, and BB performed the immunohistochemistry. MT, NL, DN, BD, and AZ investigated the QPCR analysis. AZ investigated and LP supervised the miRNA analysis. ZH, AH, GS, AK, BB, AZ, BD, AC, and MT performed the software analysis. MD, LP, LV, BP, and MS supervised the work. MD, LV, BP, and MS were the grant holders. MD, ZH, MT, and MS wrote the manuscript draft. LV, LP, and BP reviewed the manuscript. All authors approved the final manuscript.

## Conflict of Interest Statement

The authors declare that the research was conducted in the absence of any commercial or financial relationships that could be construed as a potential conflict of interest.

## References

[B1] Abbott N. J. Rönnbäck L. Hansson E. (2006). Astrocyte-endothelial interactions at the blood-brain barrier. *Nat. Rev. Neurosci.* 7 41–53. 10.1038/nrn1824 16371949

[B2] Antonios N. Angiolillo D. J. Silliman S. (2008). Hypertriglyceridemia and ischemic stroke. *Eur. Neurol.* 60 269–278. 10.1159/000157880 18824854

[B3] Armulik A. Genové G. Mäe M. Nisancioglu M. H. Wallgard E. Niaudet C. (2010). Pericytes regulate the blood-brain barrier. *Nature* 468 557–561. 10.1038/nature09522 20944627

[B4] Bandopadhyay R. Orte C. Lawrenson J. G. Reid A. R. De Silva S. Allt G. (2001). Contractile proteins in pericytes at the blood-brain and blood-retinal barriers. *J. Neurocytol.* 30 35–44. 10.1023/A:1011965307612 11577244

[B5] Barry S. O. Marcel R. T. Nelson G. Victor B. Tadeusz M. Thomas F. L. (1998). Reduced endothelial nitric oxide synthase expression and production in human atherosclerosis. *Circulation* 97 2494–2498. 10.1161/01.CIR.97.25.2494 9657467

[B6] Beauchesne E. Desjardins P. Butterworth R. F. Hazell A. S. (2010). Up-regulation of caveolin-1 and blood-brain barrier breakdown are attenuated by N-acetylcysteine in thiamine deficiency. *Neurochem. Int.* 57 830–837. 10.1016/j.neuint.2010.08.022 20816907

[B7] Bereczki E. Bernát G. Csont T. Ferdinandy P. Scheich H. Sántha M. (2008). Overexpression of human apolipoprotein B-100 induces severe neurodegeneration in transgenic mice. *J. Proteome Res.* 7 2246–2252. 10.1021/pr7006329 18473452

[B8] Betsholtz C. (2015). Lipid transport and human brain development. *Nat. Genet.* 47 699–701. 10.1038/ng.3348 26111510

[B9] Bjelik A. Bereczki E. Gonda S. Juhasz A. Rimanoczy A. Zana M. (2006). Human apoB overexpression and a high-cholesterol diet differently modify the brain APP metabolism in the transgenic mouse model of atherosclerosis. *Neurochem. Int.* 49 393–400. 10.1016/j.neuint.2006.01.026 16546298

[B10] Bowman G. L. Kaye J. A. Quinn J. F. (2012). Dyslipidemia and blood-brain barrier integrity in Alzheimer’s disease. *Curr. Gerontol. Geriatr. Res.* 2012:184042. 10.1155/2012/184042 22654903 PMC3359662

[B11] Burgess B. L. McIsaac S. A. Naus K. E. Chan J. Y. Tansley G. H. K. Yang J. (2006). Elevated plasma triglyceride levels precede amyloid deposition in Alzheimer’s disease mouse models with abundant A beta in plasma. *Neurobiol. Dis.* 24 114–127. 10.1016/j.nbd.2006.06.007 16899370

[B12] Buttery L. D. Chester A. H. Springall D. R. Borland J. A. Michel T. Yacoub M. H. (1996). Explanted vein grafts with an intact endothelium demonstrate reduced focal expression of endothelial nitric oxide synthase specific to atherosclerotic sites. *J. Pathol.* 179 197–203. 10.1002/(SICI)1096-9896(199606)179:2<197::AID-PATH587>3.0.CO;2-D 8758213

[B13] Callow M. J. Stoltzfus L. J. Lawn R. M. Rubin E. M. (1994). Expression of human apolipoprotein-B and assembly of lipoprotein (A) in transgenic mice. *Proc. Natl. Acad. Sci. U.S.A.* 91 2130–2134. 10.1073/pnas.91.6.2130 8134359 PMC43323

[B14] Caputo V. Sinibaldi L. Fiorentino A. Parisi C. Catalanotto C. Pasini A. (2011). Brain derived neurotrophic factor (BDNF) expression is regulated by microRNAs miR-26a and miR-26b allele-specific binding. *PLoS One* 6:e28656. 10.1371/journal.pone.0028656 22194877 PMC3237476

[B15] Caramelli P. Nitrini R. Maranhao R. Lourenco A. C. Damasceno M. C. Vinagre C. (1999). Increased apolipoprotein B serum concentration in Alzheimer’s disease. *Acta Neurol. Scand.* 100 61–63. 10.1111/j.1600-0404.1999.tb00724.x 10416513

[B16] Chen M. Masaki T. Sawamura T. (2002). LOX-1, the receptor for oxidized low-density lipoprotein identified from endothelial cells: implications in endothelial dysfunction and atherosclerosis. *Pharmacol. Ther.* 95 89–100. 10.1016/S0163-7258(02)00236-X 12163130

[B17] Cirrito J. R. Deane R. Fagan A. M. Spinner M. L. Parsadanian M. Finn M. B. (2005). P-glycoprotein deficiency at the blood-brain barrier increases amyloid-beta deposition in an Alzheimer disease mouse model. *J. Clin. Invest.* 115 3285–3290. 10.1172/JCI25247 16239972 PMC1257538

[B18] Cogswell J. P. Ward J. Taylor I. A. Waters M. Shi Y. Cannon B. (2008). Identification of miRNA changes in Alzheimer’s disease brain and CSF yields putative biomarkers and insights into disease pathways. *J. Alzheimers Dis.* 14 27–41. 10.3233/JAD-2008-14103 18525125

[B19] Conte C. Roscini L. Sardella R. Mariucci G. Scorzoni S. Beccari T. (2017). Toll like receptor 4 affects the cerebral biochemical changes induced by mptp treatment. *Neurochem. Res.* 42 493–500. 10.1007/s11064-016-2095-6 28108849

[B20] Croop J. M. Raymond M. Haber D. Devault A. Arceci R. J. Gros P. (1989). The three mouse multidrug resistance (mdr) genes are expressed in a tissue-specific manner in normal mouse tissues. *Mol. Cell. Biol.* 9 1346–1350. 10.1128/MCB.9.3.1346 2471060 PMC362730

[B21] Csont T. Bereczki E. Bencsik P. Fodor G. Görbe A. Zvara A. (2007). Hypercholesterolemia increases myocardial oxidative and nitrosative stress thereby leading to cardiac dysfunction in APOB-100 transgenic mice. *Cardiovasc. Res.* 76 100–109. 10.1016/j.cardiores.2007.06.006 17658498

[B22] Daneman R. Zhou L. Agalliu D. Cahoy J. D. Kaushal A. Barres B. A. (2010). The mouse blood-brain barrier transcriptome: a new resource for understanding the development and function of brain endothelial cells. *PLoS One* 5:e13741. 10.1371/journal.pone.0013741 21060791 PMC2966423

[B23] De La Torre J. C. Fortin T. Park G. A. S. Butler K. S. Kozlowski B. A. Pappas B. A. (1992). Chronic cerebrovascular insufficiency induces dementia-like deficits in aged rats. *Brain Res.* 582 186–195. 10.1016/0006-8993(92)90132-S 1327402

[B24] De la Torre J. C. Mussivand T. (1993). Can disturbed brain microcirculation cause Alzheimer’s disease? *Neurol. Res.* 15 146–153. 8103579 10.1080/01616412.1993.11740127

[B25] Deane R. Du Yan S. Submamaryan R. K. LaRue B. Jovanovic S. Hogg E. (2003). RAGE mediates amyloid-beta peptide transport across the blood-brain barrier and accumulation in brain. *Nat. Med.* 9 907–113. 10.1038/nm890 12808450

[B26] Deli M. A. Abrahám C. S. Kataoka Y. Niwa M. (2005). Permeability studies on *in vitro* blood-brain barrier models: physiology, pathology, and pharmacology. *Cell. Mol. Neurobiol.* 25 59–127. 10.1007/s10571-004-1377-8 15962509 PMC11529645

[B27] Deracinois B. Pottiez G. Chafey P. Teerlink T. Camoin L. Davids M. (2013). Glial-cell-mediated re-induction of the blood-brain barrier phenotype in brain capillary endothelial cells: a differential gel electrophoresis study. *Proteomics* 13 1185–1199. 10.1002/pmic.201200166 23436736

[B28] Di Marco L. Y. Venneri A. Farkas E. Evans P. C. Marzo A. Frangi A. F. (2015). Vascular dysfunction in the pathogenesis of Alzheimer’s disease-A review of endothelium-mediated mechanisms and ensuing vicious circles. *Neurobiol. Dis. Rev.* 82 593–606. 10.1016/j.nbd.2015.08.01 26311408

[B29] Ehehalt R. Keller P. Haass C. Thiele C. Simons K. (2003). Amyloidogenic processing of the Alzheimer beta-amyloid precursor protein depends on lipid rafts. *J. Cell. Biol.* 160 113–123. 10.1083/jcb.200207113 12515826 PMC2172747

[B30] Eiselein L. Wilson D. W. Lamé M. W. Rutledge J. C. (2007). Lipolysis products from triglyceride-rich lipoproteins increase endothelial permeability, perturb zonula occludens-1 and F-actin, and induce apoptosis. *Am. J. Physiol. Heart Circ. Physiol.* 292 H2745–H2753. 10.1152/ajpheart.00686.2006 17259442

[B31] Farkas A. S. Acsai K. Nagy N. Tóth A. Fülöp F. Seprényi G. (2008). Na ( + )/Ca (2 + ) exchanger inhibition exerts a positive inotropic effect in the rat heart, but fails to influence the contractility of the rabbit heart. *Br. J. Pharmacol.* 154 93–104. 10.1038/bjp.2008.83 18332852 PMC2438973

[B32] Gao Q. Fan Y. Mu L. Y. Ma L. Song Z. Q. Zhang Y. N. (2015). S100B and ADMA in cerebral small vessel disease and cognitive dysfunction. *J. Neurol. Sci.* 354 27–32. 10.1016/j.jns.2015.04.031 25990800

[B33] Gosselet F. Saint-Pol J. Candela P. Fenart L. (2013). Amyloid-β peptides, Alzheimer’s disease and the blood-brain barrier. *Curr. Alzheimer Res.* 10 1015–1033. 10.2174/15672050113106660174 24156262

[B34] Gundersen H. J. Osterby R. (1981). Optimizing sampling efficiency of stereological studies in biology: or ‘do more less well!’. *J. Microsc.* 121 65–73. 10.1111/j.1365-2818.1981.tb01199.x 7014910

[B35] Haley M. J. Lawrence C. B. (2017). The blood-brain barrier after stroke: structural studies and the role of transcytotic vesicles. *J. Cereb. Blood Flow Metab.* 37 456–470. 10.1177/0271678X16629976 26823471 PMC5322831

[B36] Halstrom A. MacDonald E. Neil C. Arendts G. Fatovich D. Fitzgerald M. (2017). Elevation of oxidative stress indicators in a pilot study of plasma following traumatic brain injury. *J. Clin. Neurosci.* 35 104–108. 10.1016/j.jocn.2016.09.006 27697434

[B37] Ito M. Bolati K. Kinjo T. Ichimura K. Furuta A. McLoughlin D. M. (2017). Electroconvulsive stimulation transiently enhances the permeability of the rat blood-brain barrier and induces astrocytic changes. *Brain Res. Bull.* 128 92–97. 10.1016/j.brainresbull.2016.11.011 27915091

[B38] Kacem K. Sercombe C. Hammami M. Vicaut E. Sercombe R. (2006). Sympathectomy causes aggravated lesions and dedifferentiation in large rabbit atherosclerotic arteries without involving nitric oxide. *J. Vasc. Res.* 43 289–305. 10.1159/000093010 16651846

[B39] Kapasi A. Schneider J. A. (2016). Vascular contributions to cognitive impairment, clinical Alzheimer’s disease, and dementia in older persons. *Biochim. Biophys. Acta* 1862 878–886. 10.1016/j.bbadis.2015.12.023 26769363 PMC11062590

[B40] Kisler K. Nelson A. R. Rege S. V. Ramanathan A. Wang Y. Ahuja A. (2017). Pericyte degeneration leads to neurovascular uncoupling and limits oxygen supply to brain. *Nat. Neurosci.* 20 406–416. 10.1038/nn.4489 28135240 PMC5323291

[B41] Klafke J. Z. Porto F. G. Batista R. Bochi G. V. Moresco R. N. da Luz P. L. (2015). Association between hypertriglyceridemia and protein oxidation and proinflammatory markers in normocholesterolemic and hypercholesterolemic individuals. *Clin. Chim. Acta* 448 50–57. 10.1016/j.cca.2015.06.013 26115893

[B42] Kuo Y. M. Emmerling M. R. Bisgaier C. L. Essenburg A. D. Lampert H. C. Drumm D. (1998). Elevated low-density lipoprotein in Alzheimer’s disease correlates with brain A beta 1-42 levels. *Biochem. Biophys. Res. Commun.* 252 711–715. 10.1006/bbrc.1998.9652 9837771

[B43] Lane-Donovan C. Wong W. M. Durakoglugil M. S. Wasser C. R. Jiang S. Xian X. (2016). Genetic restoration of plasma ApoE improves cognition and partially restores synaptic defects in ApoE-deficient mice. *J. Neurosci.* 36 10141–10150. 10.1523/JNEUROSCI.1054-16.2016 27683909 PMC5039258

[B44] Lee L. L. Aung H. H. Wilson D. W. Anderson S. E. Rutledge J. C. Rutkowsky J. M. (2017). Triglyceride-rich lipoprotein lipolysis products increase blood-brain barrier transfer coefficient and induce astrocyte lipid droplets and cell stress. *Am. J. Physiol. Cell Physiol.* 312 C500–C516. 10.1152/ajpcell.00120.2016 28077357 PMC5407020

[B45] Lénárt N. Szegedi V. Juhász G. Kasztner A. Horváth J. Bereczki E. (2012). Increased tau phosphorylation and impaired presynaptic function in hypertriglyceridemic ApoB-100 transgenic mice. *PLoS One* 7:e46007. 10.1371/journal.pone.0046007 23029362 PMC3454377

[B46] Lénárt N. Walter F. R. Bocsik A. Sántha P. Tóth M. E. Harazin A. (2015). Cultured cells of the blood-brain barrier from apolipoprotein B-100 transgenic mice: effects of oxidized low-density lipoprotein treatment. *Fluids Barriers CNS.* 12:17. 10.1186/s12987-015-0013-y 26184769 PMC4504453

[B47] Li B. Sun H. (2011). MiR-26a promotes neurite outgrowth by repressing PTEN expression. *Physiol. Genomics* 43 521–528. 10.3892/mmr.2013.1534 23783805

[B48] Li L. Cao D. Garber D. W. Kim H. Fukuchi K. (2003). Association of aortic atherosclerosis with cerebral beta-amyloidosis and learning deficits in a mouse model of Alzheimer’s disease. *Am. J. Pathol.* 163 2155–2164. 10.1016/S0002-9440(10)63572-9 14633589 PMC1892402

[B49] Löffler T. Flunkert S. Havas D. Sántha M. Hutter-Paier B. Steyrer E. (2013). Impact of APOB-100 expression on cognition and brain pathology in wild-type and hAPPsl mice. *Neurobiol. Aging* 34 2379–2388. 10.1016/j.neurobiolaging.2013.04.008 23643485

[B50] Lubrano V. Balzan S. (2016). Roles of LOX-1 in microvascular dysfunction. *Microvasc. Res.* 105 132–140. 10.1016/j.mvr.2016.02.006 26907636

[B51] Lutjohann D. Papassotiropoulos A. Bjorkhem I. Locatelli S. Bagli M. Oehring R. D. (2000). Plasma 24S-hydroxycholesterol (cerebrosterol) is increased in Alzheimer and vascular demented patients. *J. Lipid Res.* 41 195–198. 10681402

[B52] Lyros E. Bakogiannis C. Liu Y. Fassbender K. (2014). Molecular links between endothelial dysfunction and neurodegeneration in Alzheimer’s disease. *Curr. Alzheimers. Res.* 11 18–26. 10.2174/1567205010666131119235254 24251393

[B53] Marques F. Sousa J. C. Sousa N. Palha J. A. (2013). Blood-brain-barriers in aging and in Alzheimer’s disease. *Mol. Neurodegener.* 8:38. 10.1186/1750-1326-8-38 24148264 PMC4015275

[B54] Mattsson N. Insel P. Nosheny R. Trojanowski J. Q. Shaw L. M. Jack C. R. Jr. (2014). Effects of cerebrospinal fluid proteins on brain atrophy rates in cognitively healthy older adults. *Neurobiol. Aging* 35 614–622. 10.1016/j.neurobiolaging.2013.08.027 24094581 PMC3864623

[B55] Mayhew T. M. (1991). The new stereological methods for interpreting functional morphology from slices of cells and organs. *Exp. Physiol.* 76 639–665. 10.1113/expphysiol.1991.sp003533 1742008

[B56] Montagne A. Barnes S. R. Sweeney M. D. Halliday M. R. Sagare A. P. Zhao Z. (2015). Blood-brain barrier breakdown in the aging human hippocampus. *Neuron* 85 296–302. 10.1016/j.neuron.2014.12.032 25611508 PMC4350773

[B57] Nag S. Venugopalan R. Stewart D. J. (2007). Increased caveolin-1 expression precedes decreased expression of occludin and claudin-5 during blood-brain barrier breakdown. *Acta Neuropathol.* 114 459–469. 10.1007/s00401-007-0274-x 17687559

[B58] Nelson A. R. Sweeney M. D. Sagare A. P. Zlokovic B. V. (2016). Neurovascular dysfunction and neurodegeneration in dementia and Alzheimer’s disease. *Biochim. Biophys. Acta* 1862 887–900. 10.1016/j.bbadis.2015.12.016 26705676 PMC4821735

[B59] Nicolakakis N. Hamel E. (2011). Neurovascular function in Alzheimer’s disease patients and experimental models. *J. Cereb. Blood Flow Metab.* 31 1354–1370. 10.1038/jcbfm.2011.43 21468088 PMC3130325

[B60] Patterson C. E. Rhoades R. A. Garcia J. G. (1992). Evans blue dye as a marker of albumin clearance in cultured endothelial monolayer and isolated lung. *J. Appl. Physiol.* 72 865–873. 10.1152/jappl.1992.72.3.865 1568982

[B61] Pekny M. Wilhelmsson U. Bogestål Y. R. Pekna M. (2007). The role of astrocytes and complement system in neural plasticity. *Int. Rev. Neurobiol.* 82 95–111. 10.1016/S0074-7742(07)82005-8 17678957

[B62] Puglielli L. Tanzi R. E. Kovacs D. M. (2003). Alzheimer’s disease: the cholesterol connection. *Nat. Neurosci.* 6 345–351. 10.1038/nn0403-345 12658281

[B63] Raffaitin C. H. Gin J. P. Empana J. P. Helmer C. Berr C. Tzourio C. (2009). Metabolic syndromeand risk for incident Alzheimer’s disease or vascular dementia: the Three-City study. *Diabetes Care* 32 169–174. 10.2337/dc08-0272 18945929 PMC2606808

[B64] Sabbagh M. Zahiri H. R. Ceimo J. Cooper K. Gaul W. Connor D. (2004). Is there a characteristic lipid profile in Alzheimer’s disease? *J. Alzheimers Dis.* 6 585–589. 10.3233/JAD-2004-6602 15665398

[B65] Sagare A. P. Bell R. D. Zhao Z. Ma Q. Winkler E. A. Ramanathan A. (2013). Pericyte loss influences Alzheimer-like neurodegeneration in mice. *Nat. Commun.* 4:2932. 10.1038/ncomms3932 24336108 PMC3945879

[B66] Schubert W. Frank P. G. Woodman S. E. Hyogo H. Cohen D. E. Chow C. W. (2002). Microvascular hyperpermeability in caveolin-1 (-/-) knock-out mice. Treatment with a specific nitric-oxide synthase inhibitor, L-NAME, restores normal microvascular permeability in Cav-1 null mice. *J. Biol. Chem.* 277 40091–40098. 10.1074/jbc.M205948200 12167625

[B67] Shibata M. Yamada S. Kumar S. R. Calero M. Bading J. Frangione B. (2000). Clearance of Alzheimer’s amyloid-beta (1-40) peptide from brain by LDL receptor-related protein-1 at the blood-brain barrier. *J. Clin. Invest.* 106 1489–1499. 10.1172/JCI10498 11120756 PMC387254

[B68] Shoshani T. Zhang S. Dey S. Pastan I. Gottesman M. M. (1998). Analysis of random recombination between human MDR1 and mouse mdr1a cDNA in a pHaMDR-dihydrofolate reductase bicistronic expression system. *Mol. Pharmacol.* 54 623–630. 9765504

[B69] Song L. Ge S. Pachter J. S. (2007). Caveolin-1 regulates expression of junction-associated proteins in brain microvascular endothelial cells. *Blood* 109 1515–1523. 10.1182/blood-2006-07-034009 17023578 PMC1794065

[B70] Stancel N. Chen C. C. Ke L. Y. Chu C. S. Lu J. Sawamura T. (2016). Interplay between CRP, atherogenic LDL, and LOX-1 and its potential role in the pathogenesis of atherosclerosis. *Clin. Chem.* 62 320–327. 10.1373/clinchem.2015.243923 26607724

[B71] Stapleton P. A. Goodwill A. G. James M. E. Brock R. W. Frisbee J. C. (2010). Hypercholesterolemia and microvascular dysfunction: interventional strategies. *J. Inflamm.* 7:54. 10.1186/1476-9255-7-54 21087503 PMC2996379

[B72] Süle Z. Mracskó E. Bereczki E. Sántha M. Csont T. Ferdinandy P. (2009). Capillary injury in the ischemic brain of hyperlipidemic, apolipoprotein B-100 transgenic mice. *Life Sci.* 84 935–939. 10.1016/j.lfs.2009.04.011 19409916

[B73] Varendi K. Kumar A. Härma M. A. Andressoo J. O. (2014). miR-1, miR-10b, miR-155, and miR-191 are novel regulators of BDNF. *Cell. Mol. Life Sci.* 71 4443–4456. 10.1007/s00018-014-1628-x 24804980 PMC4207943

[B74] Veszelka S. Pásztói M. Farkas A. E. Krizbai I. Ngo T. K. Niwa M. (2007). Pentosan polysulfate protects brain endothelial cells against bacterial lipopolysaccharide-induced damages. *Neurochem. Int.* 50 219–228. 10.1016/j.neuint.2006.08.006 16997427

[B75] Veszelka S. Tóth A. Walter F. R. Tóth A. E. Gróf I. Mészáros M. (2018). Comparison of a rat primary cell-based blood-brain barrier model with epithelial and brain endothelial cell lines: gene expression and drug transport. *Front. Mol. Neurosci.* 11:166. 10.3389/fnmol.2018.00166 29872378 PMC5972182

[B76] Veszelka S. Tóth A. E. Walter F. R. Datki Z. Mózes E. Fülöp L. (2013). Docosahexaenoic acid reduces amyloid-β induced toxicity in cells of the neurovascular unit. *J. Alzheimers Dis.* 34 487–501. 10.3233/JAD-120163 23645098

[B77] Veszelka S. Urbányi Z. Pázmány T. Németh L. Obál I. Dung N. T. (2003). Human serum amyloid P component attenuates the bacterial lipopolysaccharide-induced increase in blood-brain barrier permeability in mice. *Neurosci. Lett.* 352 57–60. 10.1016/j.neulet.2003.08.028 14615049

[B78] Vogel C. Marcotte E. M. (2012). Insights into the regulation of protein abundance from proteomic and transcriptomic analyses. *Nat. Rev. Genet.* 13 227–232. 10.1038/nrg3185 22411467 PMC3654667

[B79] Wang H. Zhu H. Q. Wang F. Zhou Q. Gui S. Y. Wang Y. (2013). MicroRNA-1 prevents high-fat diet-induced endothelial permeability in apoE knock-out mice. *Mol. Cell. Biochem.* 378 153–159. 10.1007/s11010-013-1606-x 23467882 PMC3634980

[B80] Willis C. L. (2012). Imaging *in vivo* astrocyte/endothelial cell interactions at the blood-brain barrier. *Methods Mol. Biol.* 814 515–529. 10.1007/978-1-61779-452-0_34 22144329

[B81] Winkler E. A. Bell R. D. Zlokovic B. V. (2011). Central nervous system pericytes in health and disease. *Nat. Neurosci.* 14 1398–1405. 10.1038/nn.2946 22030551 PMC4020628

[B82] Winkler E. A. Nishida Y. Sagare A. P. Rege S. V. Bell R. D. Perlmutter D. (2015). GLUT1 reductions exacerbate Alzheimer’s disease vasculo-neuronal dysfunction and degeneration. *Nat. Neurosci.* 18 521–530. 10.1038/nn 25730668 PMC4734893

[B83] Wolburg H. Noell S. Wolburg-Buchholz K. Mack A. Fallier-Becker P. (2009). Agrin, aquaporin-4, and astrocyte polarity as an important feature of the blood-brain barrier. *Neuroscientist* 15 180–193. 10.1177/1073858408329509 19307424

[B84] Won D. Zhu S. N. Chen M. Teichert A. M. Fish J. E. Matouk C. C. (2007). Relative reduction of endothelial nitric-oxide synthase expression and transcription in atherosclerosis-prone regions of the mouse aorta and in an *in vitro* model of disturbed flow. *Am. J. Pathol.* 171 1691–1704. 10.2353/ajpath.2007.060860 17982133 PMC2043529

[B85] Wu Z. Guo H. Chow N. Sallstrom J. Bell R. D. Deane R. (2005). Role of the MEOX2 homeobox gene in neurovascular dysfunction in Alzheimer disease. *Nat. Med.* 11 959–965. 10.1038/nm1287 16116430

[B86] Zhao Z. Nelson A. R. Betsholtz C. Zlokovic B. V. (2015). Establishment and dysfunction of the blood-brain barrier. *Cell* 163 1064–1078. 10.1016/j.cell.2015.10.06 26590417 PMC4655822

[B87] Zhao Z. Zlokovic B. V. (2014). Blood-brain barrier: a dual life of MFSD2A? *Neuron* 82 728–730. 10.1016/j.neuron.2014.05.012 24853933 PMC4114515

[B88] Zipser B. D. Johanson C. E. Gonzalez L. Berzin T. M. Tavares R. Hulette C. M. (2007). Microvascular injury and blood-brain barrier leakage in Alzheimer’s disease. *Neurobiol. Aging* 28 977–986. 10.1016/j.neurobiolaging.2006.05.016 16782234

[B89] Zlokovic B. V. (2008). The blood-brain barrier in health and chronic neurodegenerative disorders. *Neuron* 57 178–201. 10.1016/j.neuron.2008.01.003 18215617

[B90] Zlokovic B. V. (2011). Neurovascular pathways to neurodegeneration in Alzheimer’s disease and other disorders. *Nat. Rev. Neurosci.* 3 723–738. 10.1038/nrn3114 22048062 PMC4036520

