# Supplementary material for: Cerebrovascular Pathology in Hypertriglyceridemic APOB-100 Transgenic Mice
Source: Front Cell Neurosci. 2018 Oct 25;12:380. doi: 10.3389/fncel.2018.00380 (PMC6209654; doi:10.3389/fncel.2018.00380)
Supplement: Supplementary file 1 [file Data_Sheet_1.docx]

**Supplementary material**

**Cerebrovascular pathology in hypertriglyceridemic APOB-100 transgenic mice**

Zsófia Hoyk^1+^, Melinda E. Tóth^2+^, Nikolett Lénárt^2#^, Dóra Nagy^2^, Brigitta Dukay^2^, Alexandra Csefová^2^, Ágnes Zvara^3^, György Seprényi^4^, András Kincses^1^, Fruzsina R. Walter^1^, Szilvia Veszelka^1^, Judit Vígh^1^, Beáta Barabási^1^, András Harazin^1^, Ágnes Kittel^5^, László G. Puskás^3^, Botond Penke^6^, László Vígh^2^, Mária A. Deli^1*^ and Miklós Sántha^2*^

**Table S1.** Age and number of animals used in this study.

| **Experiment** | **Age of animals (months)** | **Number of animals/group** |
| --- | --- | --- |
| Serum triglyceride measurement | 7, 9 and 12 | 5 |
| BBB permeability | 6 | 10 |
| TEM | 7 | 4 |
| Microvessel RT-PCR | 6-7 | 6 |
| Cortex, hippocampus RT-PCR | 6-7 | 6 |
| Fluorescence intensity analysis | 7-8 | 5 |
| Primary brain endothelial cell isolation | 6 | 10 |
| Primary pericyte isolation | 6-7 | 10 |
| Primary astrocyte isolation | 1 day | 2 |

**Table S2.** List of primer sequences used in this study

| **gene** | **forward** | **reverse** |
| --- | --- | --- |
| ACTB | CTAAGGCCAACCGTGAAAAG | ACCAGAGGCATACAGGGACA |
| AQP4 | CTTTCTGGAAGGCAGTCTCAG | CCACACCGAGCAAAACAAAGAT |
| BDNF | AGTCTCCAGGACAGCAAAGC | TGCAACCGAAGTATGAAATAACC |
| Cav-1 | GCGACCCCAAGCATCTCAA | ATGCCGTCGAAACTGTGTGT |
| CD40 | TTGTTGACAGCGGTCCATCTA | GCCATCGTGGAGGTACTGTTT |
| Cldn5 | ACGGGAGGAGCGCTTTAC | GTTGGCGAACCAGCAGAG |
| eNOS | GGCTGGGTTTAGGGCTGTG | CTGAGGGTGTCGTAGGTGATG |
| GAPDH | GGGTTCCTATAAATACGGACTGC | CCATTTTGTCTACGGGACGA |
| GFAP | CGGAGACGCATCACCTCTG | AGGGAGTGGAGGAGTCATTCG |
| GFAP-α | GGAGATGCGGGATGGTGAG | ACCACGTCCTTGTGCTCCTG |
| GFAP-δ | TCTCCAACCTCCAGATCCGA | TGACTTTTTGGCCTTCCCCT |
| Glut1 (Slc2a1) | GGATCCCAGCAGCAAGAAG | CCAGTGTTATAGCCGAACTGC |
| Gp91phox | AGTGCGTGTTGCTCGACAA | GCGGTGTGCAGTGCTATCAT |
| Iba-1 | ATCAACAAGCAATTCCTCGATGA | CAGCATTCGCTTCAAGGACATA |
| IL-1β | GCAACTGTTCCTGAACTCAACT | ATCTTTTGGGGTCCGTCAACT |
| iNOS | GTTCTCAGCCCAACAATACAAGA | GTGGACGGGTCGATGTCAC |
| LRP1 | ATTGAGGGCAAGATGACACA | CCAGTCTGTCCAGTACATCCAC |
| LRP2 | GATGGATTAGCCGTGGACTG | TCCGTTGACTCTTAGCATCTGA |
| Mac1 | CCATGACCTTCCAAGAGAATGC | ACCGGCTTGTGCTGTAGTC |
| MEOX2 (MOX2) | AATCTAGACCTCACTGAAAGACAGG | CTTTGACCCGCTTCCACTT |
| Mfsd2a | AGAAGCAGCAACTGTCCATTT | CTCGGCCCACAAAAAGGATAAT |
| N-cadherin | AGCGCAGTCTTACCGAAGG | TCGCTGCTTTCATACTGAACTTT |
| NF-κB | ATGGCAGACGATGATCCCTAC | TGTTGACAGTGGTATTTCTGGTG |
| NLGN1 | GGTACTTGGCTTCTTGAGCAC | AAACACAGTGATTCGCAAGGG |
| NMDAR | AGAGCCCGACCCTAAAAAGAA | CCCTCCTCCCTCTCAATAGC |
| NRXN1 | AACGGACTGATGCTTCACACA | CCTGAGTGCTGACGCAGATT |
| Ocln | CACGACAGGTGGGGAGTC | TTGATCTGAAGTGATAGGTGGATATT |
| Olr1 (LOX1) | CCTGCTGCTATGACTCTGGTC | GAGGTCAGATACCTGGCGTAA |
| P-gp (ABCb1a) | AATGTTTCGTTATGCAGGTTGGC | TGGCTCTTTTATCGGCCTCAC |
| P-gp  (ABCb1b) | AGTGGACCCAACAGTACTCTGAT | GCACCAATCCCGGTGTAATA |
| S100b | TGGTTGCCCTCATTGATGTCT | CCCATCCCCATCTTCGTCC |
| TLR-4 | ATGGCATGGCTTACACCACC | GAGGCCAATTTTGTCTCCACA |
| TNFα | CCCTCACACTCAGATCATCTTCT | GCTACGACGTGGGCTACAG |
| Vimentin | CGTCCACACGCACCTACAG | GGGGGATGAGGAATAGAGGCT |
| ZO1(Tjp1) | CGCGGAGAGAGACAAGATGT | AGCGTCACTGTGTGCTGTTC |

**Table S3** List of antibodies used in this study

| **Antibody name** | **Vendor** | **Catalogue number** | **Host organism** | **Dilution** | **Antibody registry ID** |
| --- | --- | --- | --- | --- | --- |
| Claudin-5 | Sigma | SAB 4502981 | rabbit | 1:800 | [AB_10753223](http://antibodyregistry.org/AB_10753223) |
| Occludin | ThermoFisher Scientific | 71-1500 | rabbit | 1:650 | AB_2533977 |
| Vimentin | DAKO | M0725 | mouse | 1:500 | AB_10013485 |
| P-gp | Millipore | 517310 | mouse | 1:150 | [AB_564389](http://antibodyregistry.org/AB_564389) |
| Gfap | Abcam | ab53554 | goat | 1:1000 | AB_880202 |
| Pdgfrβ | Abcam | ab32570 | rabbit | 1:200 | [AB_777165](http://antibodyregistry.org/AB_777165) |
| Lox-1 | Abcam | ab60178 | rabbit | 1:500 | [AB_943982](http://antibodyregistry.org/AB_943982) |

**Lox-1 immunohistochemistry**

Lox-1 immunoreactivity was visualized using the avidin-biotin-peroxidase method. Endogenous peroxidase activity was blocked by incubating the sections in 3% H_2_O_2_ in PBS and 20% methanol for 15 minutes Antigen retrieving was performed with 0.3% Triton X-100 treatment. Non-specific immunoreactivity was blocked with 2 % normal horse serum in PBS for 2 h at room temperature. Lox-1 primary antibody was used at 1:500 dilution **(Supplementary Table S3)**. The primary antibody was omitted in negative immunohistochemical controls. For positive immunohistochemical controls heart coronary vessels of APOB-100 transgenic mice were used. The secondary antibody was a biotinylated donkey anti-rabbit IgG (Jackson ImmunoResearch Europe Ltd., Cambridgeshire, UK) Following the incubation with the secondary antibody sections were washed in PBS and incubated in Vectastain Elite ABC reagent (Vector, 1:250, Olean, NY, USA). The reaction product was visualized by incubating the sections in 0.042 % 3,3’-diaminobenzidine and 0.002 % hydrogen peroxide in 0.1 M tris buffered saline. The immunostained sections were mounted on slides, dehydrated in an ascending series of alcohol and coverslipped with Entellan (Merck, Darmstadt, Germany). The immunoreactivity pattern was analyzed with an Olympus Vanox-T microscope (Olympus Life Science Europa GmbH, Hamburg, Germany).

**Lox-1 image analysis**

Images (representing 5 animals from each group) were analyzed with the public domain Fiji software. First, areas showing randomly selected parts of the hippocampus and cortex (three cortical and three hippocampal areas from each animal) were measured. Then, grayscale 8-bit images were converted to thresholded 1-bit images, on which the area occupied by Lox-1 immunolabeled structures was measured considering particles larger than 1 µm^2^ and with circularity values between 0-1. Next, the area fraction of Lox-1 immunoreactive structures was calculated both in the cortex and hippocampus.

**Statistical analysis of the area fraction of Lox-1 immunolabeled structures**

GraphPad Prism 5.0 software (GraphPad Software Inc.,LaJolla, CA, USA) was used for statistical analysis. Data were analyzed with two-way analysis of variance followed by Bonferroni *posthoc* test The level of statistical significance was taken as *p* < 0.05. Results are presented as means ± SEM.

**
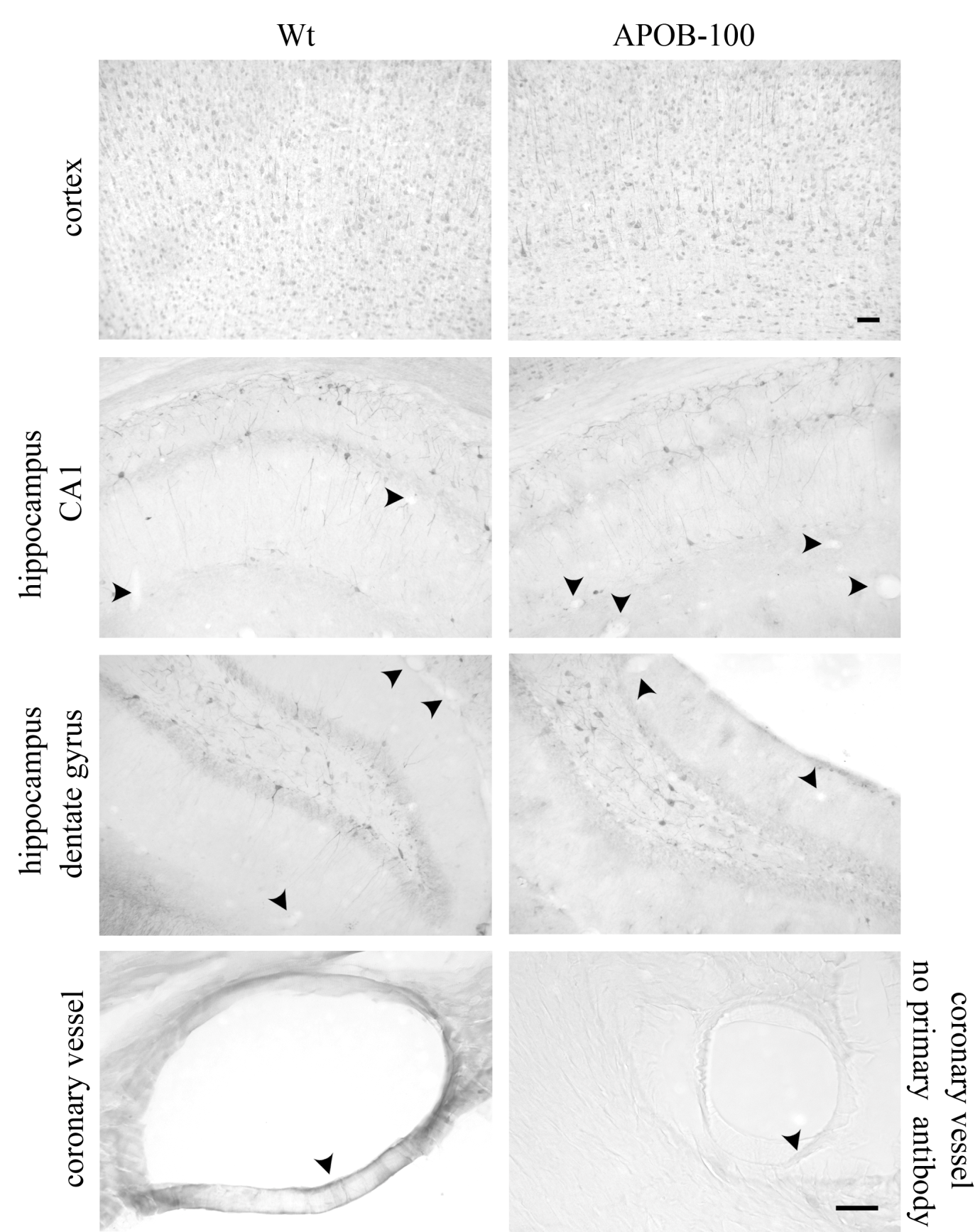
**

**Supplementary Figure S1.**

Lox-1 immunostaining in the cortex and hippocampus (CA1 region and dentate gyrus) of wild-type (Wt) and APOB-100 transgenic mice (APOB-100). Heart coronary vessels were used as positive and negative immunohistochemical controls. Black arrows indicate hippocampal vessels. Scale bars: 50 µm

**
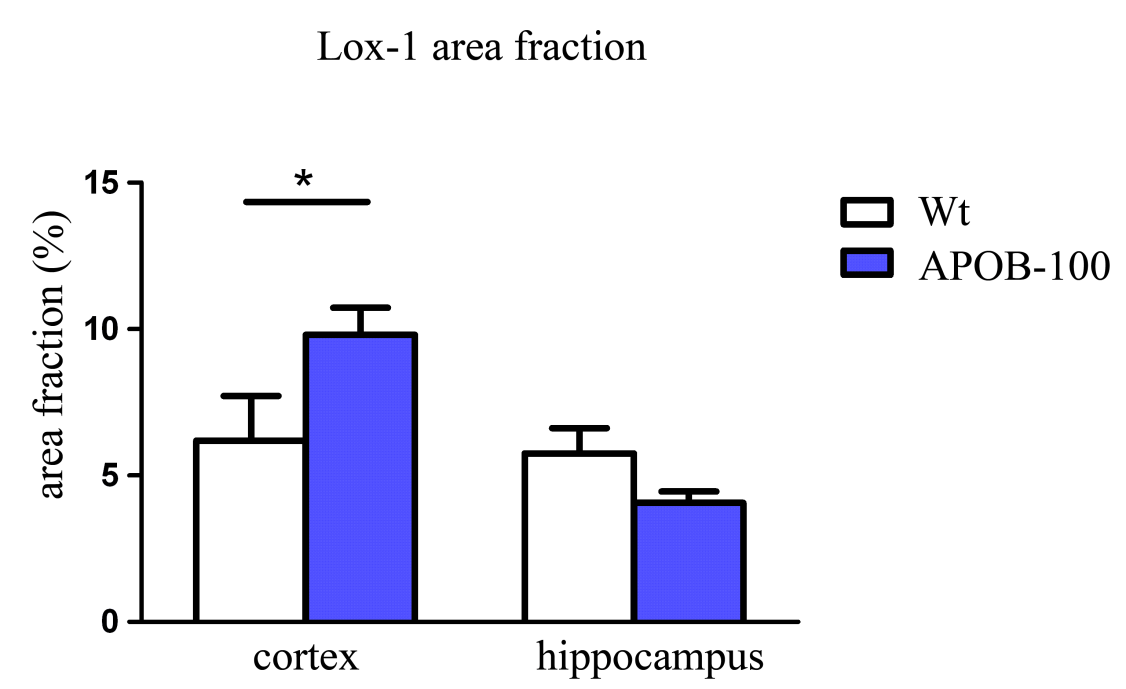
**

**Supplementary Figure S2.**

Quantification of Lox-1 immunoreactive structures in the cortex and hippocampus of Wt and APOB-100 transgenic mice.


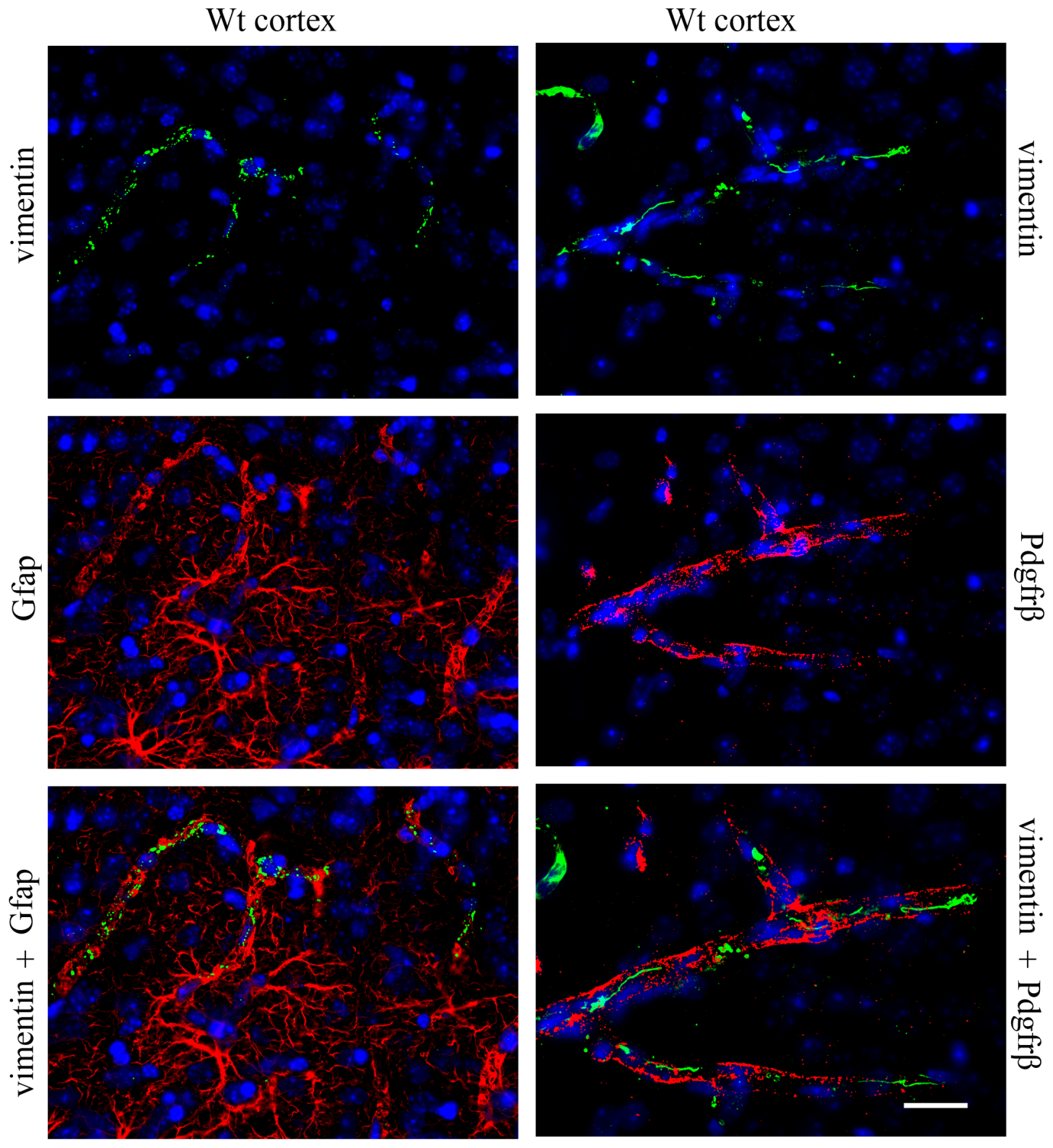


**Supplementary Figure S3.**

Vimentin localization in cortical sections of wild-type (Wt) mice, using vimentin (green) + GFAP (red) and vimentin (green) +PDGFRβ (red) double immunostainings and DAPI (blue) nuclear staining. No co-localization of vimentin was detected either with GFAP or with PDGFRβ. Scale bar: 25 µm

**Primary cell isolation for vimentin gene expression analysis**

Primary mouse brain endothelial cell isolation was performed using 6-7-month-old C57BL/6 wild type and transgenic mice based on methods established in our lab (Veszelka et al., 2013, Lénárt et al., 2015). Mouse forebrains were collected in ice-cold sterile phosphate buffered saline (PBS). Meninges were removed using sterile filter paper and the tissue was minced into 1mm^3^ pieces by scalpels and digested by enzymes (1 mg/ml collagenase type II and 15 µg/ml DNase type I, Roche, Switzerland) in Dulbecco’s modified Eagle medium (DMEM/F12, Gibco, Life Technologies, USA) at 37 °C for 50 min. Microvessels were separated from myelin by centrifugation on a 20 % bovine serum albumin (BSA)-DMEM gradient (1000 *g*, 20 min, 3 times). The collected vessels were further digested by enzymes (1 mg/ml collagenase–dispase and DNase type I in DMEM, both from Roche, Switzerland) for 35 min. Digested cerebral vessels were washed three times in cell culture medium then seeded onto Petri dishes (60 mm; Orange Scientific, Belgium) coated with collagen type IV and fibronectin (100 µg/ml each). Mouse brain endothelial cells were cultured in DMEM/F12 supplemented with plasma-derived bovine serum (15 %; First Link, UK), heparin (100 µg/ml), basic fibroblast growth factor (1 ng/ml; Roche, Switzerland), insulin (5 µg/ml), transferrin (5 µg/ml), sodium selenite (5 ng/ml), and gentamycin (50 µg/ml). Brain endothelial cells were grown under selective culture conditions in the first 4 days: culture medium contained puromycin (3 µg/ml) to eliminate P-glycoprotein negative contaminating cell types (Perriere et al., 2005). When endothelial cells reached confluency, the cells were ready for RNA isolation.

Primary cultures of mouse pericytes were prepared by the same protocol as primary mouse brain endothelial cells, except for a shorter time of the second enzymatic digestion (15 min) and the omission of the puromycin treatment. At the end of the isolation process cerebral microvessels containing pericytes beside endothelial cells were seeded into collagen type IV. coated 60 mm culture dishes (Orange Scientific, Belgium). After 4 days of culture in DMEM, 10 % fetal bovine serum (FBS), and gentamycin (50 µg/ml) attached cells reached 70 % of confluency. Pericytes were passaged into bigger, uncoated dishes (Orange Scientific, Belgium). Mouse pericyte cultures were used at second passage. Primary mouse pericytes stain positive for α-smooth muscle actin and NG2, but not for von Willebrand factor or glial fibrillary acidic protein (GFAP) (Nakagawa et al., 2009, Lénárt et al., 2015).

Primary mouse glial cells were obtained from 2-day-old wild type or APOB-100 transgenic mice (Lénárt et al., 2015). Meninges were removed by fine forceps from brains. Little pieces of cortices were minced and mechanically dissociated by pressing the tissue through a nylon mesh (40 µm, Millipore, USA). Cell clusters were seeded onto uncoated 75 cm^2^ flasks (TPP, Germany) and cultured in DMEM containing FBS (10 %; Lonza, Switzerland) and gentamycin (50 µg/ml) until 90% confluency. Glial cells were passaged only once before use.

**References**

# Lénárt, N., Walter, F. R., Bocsik, A., Sántha, P., Tóth, M. E., Harazin, A., et al. (2015). Cultured cells of the blood-brain barrier from apolipoprotein B-100 transgenic mice: effects of oxidized low-density lipoprotein treatment. *Fluids Barriers CNS.* 12:17. doi: 10.1186/s12987-015-0013-y.

Nakagawa, S., Deli, M. A., Kawaguchi, H., Shimizudani, T., Shimono, T., Kittel, A., et al. (2009). A new blood-brain barrier model using primary rat brain endothelial cells, pericytes and astrocytes. *Neurochem Int*. 54: 253-263. doi: 10.1016/j.neuint.2008.12.002

Perrière, N., Demeuse, P., Garcia, E., Regina, A., Debray, M., et al. (2005). Puromycin-based purification of rat brain capillary endothelial cell cultures. Effect on the expression of blood-brain barrier-specific properties. *J Neurochem*. 93: 279-289. doi: 10.1111/j.1471-4159.2004.03020.x

Veszelka, S., Tóth, AE., Walter, FR., Datki, Z., Mózes, E., Fülöp, L., et al. (2013) Docosahexaenoic acid reduces amyloid-β induced toxicity in cells of the neurovascular unit. *J Alzheimers Dis.* 36: 487–501. doi: 10.3233/JAD-120163


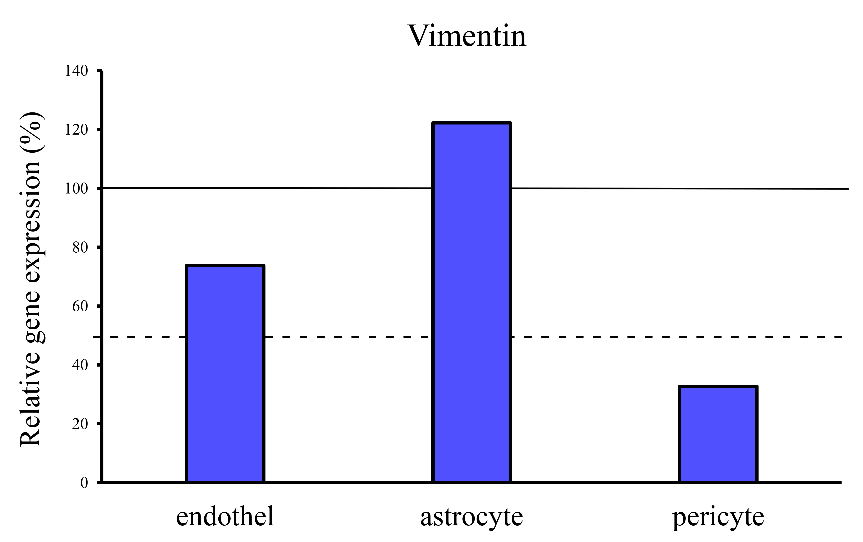


**Supplementary Figure S4.**

Analysis of vimentin gene expression level in isolated primary cells (brain capillary endothelial cells, astrocytes and pericytes), using QPCR. Continuous line indicates vimentin gene expression level in wild-type mice (100%). Dashed line indicates a 50% drop in gene expression, which is considered as the threshold of significant reduction.

**

**

**Supplementary Figure S5.**

TEM images showing microvessels in the cortex and hippocampus of Wt and APOB-100 transgenic mice. * indicates swollen astroglia endfeet surrounding microvessels. Scale bar: 1µm.

**miRNA profiling and data analysis**

Total RNA was purified from the brain of 6-month-old transgenic and wild-type animals (n=3 animals/ group) using an RNA purification kit (Macherey Nagel, Düren, Germany), according to the manufacturer’s instructions. At a final concentration of 0.8 U/μl, an RNase inhibitor (Fermentas, Lithuania) was added to the samples. RNA quantity was determined using NanoDrop 3.1.0. RNA samples were stored at –80°C before use. 100-100ng of six purified mouse total RNA samples (wild-type female cortices n=3, APOB-100 female cortices n=3) were analyzed by the NanoString custom miRNA analysis service using nCounter mouse miRNA assay kit (http://www.nanostring.com). miRNA assays were performed according to the NanoString miRNA Assay Manual. During the analysis, sequence specific fluorescently labeled bar coded probes were hybridized to the miRNAs of interest. After scanning, the nCounter detected the total counts of each bar-coded miRNA signals. Hybridizations were carried out by combining 5µl of each miRNA assay with 20µl of nCounter Reporter probes in hybridization buffer and 5µl of nCounter Capture probes for a total reaction volume of 30µl. The hybridization reaction mixtures were incubated at 65°C for approximately 16-20 hours. Raw data were collected using Nanostring’s nSolver Analysis application (NanoString Technologies, Seattle, WA, USA). Counts were normalized to the top 100-expressed miRNA, which accounts for variations in sample miRNA content and should provide the most meaningful data for sample comparison (https://www.nanostring.com/products/analysis-software/nsolver). A feature was considered “detected” if: i) the average value of the three parallel samples were above the mean level of the negative controls plus two standard deviations of the mean (meanSample>meanNegCont + 2SD) and ii) the levels of three parallel samples were significantly higher than the levels of the negative controls (two tailed two sample unequal variance Student t-test, p<0.05). After this feature quality control, p-value was determined and used to find the significant expression changes using two-tailed two-sample unequal variance Student t-test. Expression ratios with p < 0.05 and log2 ratio < -0.7 or log2 ratio > 0.7 (~1.6 fold change) were considered as repression or overexpression, respectively, in miRNA gene activity (Geiss et al., 2008).

**Reference**

Geiss, G. K., Bumgarner, R. E., Birditt, B., Dahl, T., Dowidar, N., Dunaway, D. L., et al. (2008). Direct multiplexed measurement of gene expression with color-coded probe pairs. *Nat Biotechnol*. 26, 317–325. doi: 10.1038/nbt1385

**Table S4** List of miRNAs that showed significant differential expression in ApoB-100 transgenic animals.

| **Accession number** | **ID** | **average change (log2)** | **p value** | **fold change** | **regulation** |
| --- | --- | --- | --- | --- | --- |
| mmu-miR-7a-5p | MIMAT0000677 | -0.98 | 0.0250 | 1.98 | down |
| mmu-miR-7b-5p | MIMAT0000678 | -0.83 | 0.0127 | 1.77 | down |
| mmu-miR-187-3p | MIMAT0000216 | -0.81 | 0.0176 | 1.76 | down |
| mmu-miR-1a-3p | MIMAT0000123 | -0.74 | 0.0055 | 1.67 | down |
| mmu-miR-669g | MIMAT0005832 | 0.71 | 0.0096 | 1.63 | up |
| mmu-miR-222-3p | MIMAT0000670 | 0.73 | 0.0013 | 1.66 | up |
| mmu-miR-708-5p | MIMAT0004828 | 0.74 | 0.0057 | 1.67 | up |
| mmu-miR-26a-5p | MIMAT0000533 | 0.74 | 0.0010 | 1.67 | up |
| mmu-miR-1898 | MIMAT0007875 | 0.74 | 0.0004 | 1.68 | up |
| mmu-miR-500-3p | MIMAT0003507 | 0.78 | 0.0000 | 1.71 | up |


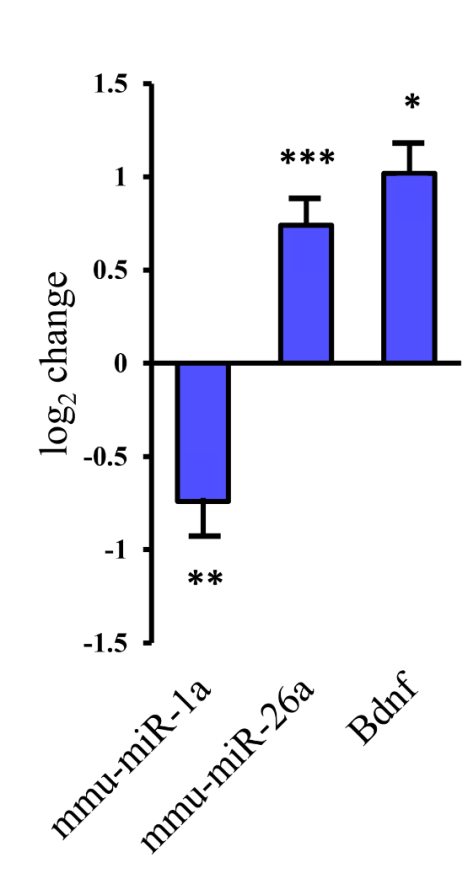


**Supplementary Figure S6.**

miRNA expression alterations (miR-1a and 268 miR-26a) and their target expression level (Bdnf) in the cortex of APOB-100 transgenic mice. P***<0.001, P**<0.01, P*<0.05, compared with Wt mice.
